# Optimization-based framework with flux balance analysis (FBA) and metabolic pathway analysis (MPA) for identifying metabolic objective functions

**DOI:** 10.1371/journal.pcbi.1013635

**Published:** 2025-10-27

**Authors:** Ching-Mei Wen, Eleftherios Papoutsakis, Marianthi Ierapetritou

**Affiliations:** Department of Chemical and Bio-molecular Engineering, University of Delaware, Newark, Delaware, United States of America; Teesside University, UNITED KINGDOM OF GREAT BRITAIN AND NORTHERN IRELAND

## Abstract

Metabolic network modeling, especially Flux Balance Analysis (FBA), plays a critical role in systems biology by providing insights into cellular behaviors. Although FBA is the main tool for predicting flux distributions, it can face challenges capturing flux variations under different conditions. Selecting an appropriate objective function is therefore important for accurately representing system performance. To address this, we introduce a novel framework (e.g., **TIObjFind**) that imposes Metabolic Pathway Analysis (MPA) with Flux Balance Analysis (FBA) to analyze adaptive shifts in cellular responses throughout different stages of a biological system. This framework determines Coefficients of Importance (CoIs) that quantify each reaction’s contribution to an objective function, aligning optimization results with experimental flux data. By examining Coefficients of Importance, **TIObjFind** enhances the interpretability of complex metabolic networks and provides insights into adaptive cellular responses.

## Author summary

Cells adjust their metabolism dynamically in response to environmental changes, and computational models help us understand these adaptations. Flux Balance Analysis is a widely used approach to predict cellular metabolism, but its accuracy relies on selecting an appropriate metabolic objective function. Here, we introduce **TIObjFind**, a data-driven optimization framework that helps computational models better predict how metabolic networks prioritize reactions under different conditions. **TIObjFind** identifies key reactions by assigning Coefficients of Importance, which indicate the contribution to cellular objectives. Two case studies illustrate the application of **TIObjFind**. The first case study focuses on the fermentation of glucose by *Clostridium acetobutylicum*, where the proposed method is used to determine pathway-specific weighting factors. By applying different weighting strategies, we assess the influence of Coefficients of Importance on flux predictions and demonstrate their impact on reducing prediction errors while improving the alignment with experimental data. The second case study examines a multi-species isopropanol-butanol-ethanol (IBE) system comprising *C. acetobutylicum*, and *C. ljungdahlii*. In this case, the weights (or Coefficients of Importance) are used as hypothesis coefficients within the objective function to assess cellular performance. Application of the proposed approach demonstrates a good match with observed experimental data and capturing stage-specific metabolic objectives.

## 1. Introduction

Metabolic networks have been extensively used in various fields, such as drug discovery [1], microbial strain improvement [2], systems biology [3], disease diagnosis [4], and understanding evolutionary dynamics [5]. Rather than limiting research to isolated reactions or pathways, a comprehensive analysis of these networks offers insights into the broader interplay of cellular functions. As such, metabolic network analysis is analogous to examining the full circuitry of a cell, charting how nutrients, metabolites, and energy flow and interact. Resources, such as KEGG [6] and EcoCyc [7] offer extensive insights into biological pathways, genomic, chemical, and network information, serving as foundational databases for researchers.

While metabolic networks offer extensive insights, the shifts and stimulations of the environment leading to different regulatory approaches in microorganisms in response to these changes might not effectively be tracked [8]. The shifts and stimulations of the surrounding conditions may be associated with the secretion of solvents [9–11], nutrient availability [12], and/or inherent system properties. Additionally, the accuracy of the models often depends on the availability of well-annotated genomic information, which typically does not account for changes in the surrounding conditions [12]. As a result, several metabolic network modeling methods [3,13–19] and tools [20] have been developed to address these issues. Flux Balance Analysis (FBA) [3] and its dynamic extension, Dynamic Flux Balance Analysis (dFBA) [19], are central tools in systems biology for analyzing cellular metabolism. FBA primarily focuses on steady-state conditions, calculating optimal metabolic flux distributions that align with specific cellular objectives. Common objectives include the synthesis of biomass, production of primary and secondary metabolites, ATP generation, and regulating growth rates. As more complex networks and higher-order systems are explored, alternative objectives may offer improved accuracy in predicting the phenotypical characteristics of cells. Previously, *in silico* frameworks have been developed to identify the most likely objective function for such systems making it particularly suitable for studying shifts and perturbations in environmental conditions over time [21–23]. For example, regulatory flux balance analysis (rFBA) has been developed to explicitly account for the impact of gene regulation on metabolic states by integrating Boolean logic-based rules with FBA, thereby constraining reaction activity based on gene expression states and environmental signals [24]. Flexible implementations such as FlexFlux have extended this concept by combining qualitative regulatory networks with constraint-based modeling at genome scale, without requiring detailed kinetic parameters [25].

In addition to these extensions, the **ObjFind** framework builds upon traditional FBA by introducing Coefficients of Importance (CoIs) [22], which quantify each flux’s additive contribution to a chosen objective function, aiming to align model predictions with observed experimental flux data, $v_{j}^{exp}$. By maximizing a weighted sum of fluxes with coefficient, *c*_*j*_, while minimizing the sum of squared deviations from experimental data (e.g., **ObjFind** can be thought of as a scalarization of a multi-objective problem), **ObjFind** enables the interpretation of experimental fluxes in terms of optimized metabolic objectives. Here, each coefficient *c*_*j*_ represents the relative importance of a reaction, scaling these coefficients so their sum equals one. A higher *c*_*j*_ suggests that a reaction flux aligns closely with its maximum potential, indicating that the experimental flux data may be directed toward optimal values for specific pathways. Although the **ObjFind** framework shows that maximizing a weighted combination of fluxes can capture the performance of a given set of observed experimental data, it assigns weights across all metabolites and has the potential for overfitting to particular conditions. Furthermore, experimental work such as isotopomer analysis is required for $v_{j}^{exp}$ determination.

Building on **ObjFind**, we present a novel framework to identify hypothesized objective functions for biological systems. This approach uses the stoichiometry of biochemical networks and experimental flux data to construct a flux-dependent weighted reaction graph [26], quantifying each flux’s additive contribution (or Coefficients of Importance) with Metabolic Pathway Analysis (MPA) [27,28]. The graph integrates the impact of environmental perturbations by using FBA solutions under varying cellular conditions. The framework proposed here, referred to as **TIObjFind** (Topology-Informed Objective Find), solves an optimization problem that minimizes the difference between predicted fluxes, derived from a potential cellular objective (e.g., yield analysis), and experimental data of observed external compounds. Using the calculated fluxes to construct a flux-dependent weighted reaction graph, **TIObjFind** applies a path-finding algorithm to analyze Coefficients of Importance between selected start reactions (e.g., glucose uptake as a primary metabolic input) and target reactions (e.g., product secretion). By focusing on specific pathways rather than the entire network, this method highlights critical connections, the interpretability of dense metabolic networks can be improved.

The topology-informed method selectively evaluates fluxes in key pathways, enhancing interpretability and adaptability. This integration of MPA with FBA enables us to capture metabolic flexibility, offering insights into cellular responses under environmental changes and providing a systematic mathematical framework for modeling complex, adaptive networks. Based on the proposed **TIObjFind** framework, we examine: (i) Analyzing the difference in Coefficients of Importance across different stages of a biological system to reveal shifting metabolic priorities; (ii) Identifying the objective function that best aligns with experimental flux data.

## 2. Results and discussion

### 2.1. Overview of Topology-Informed Objective Find (TIObjFind)

From a practical perspective, researchers often perform FBA with an objective function that assumes a single reaction, such as biomass maximization or metabolite production, as the exclusive optimization goal [29]. Without considering how alternative pathways contribute to overall network function, these static objectives may not always align with observed experimental flux data, particularly under changing environmental conditions. To address this limitation, we introduce **TIObjFind**, a novel framework that integrates Metabolic Pathway Analysis (MPA) with FBA to systematically infer metabolic objectives from data. **TIObjFind** distributes importance to metabolic pathways using Coefficients of Importance, utilizing network topology and pathway structure to analyze metabolic behavior across different system states.

**TIObjFind** has three key steps: (i) it reformulates the objective function selection as an optimization problem that minimizes difference between predicted and experimental fluxes while maximizing an inferred metabolic goal, (ii) it maps FBA solutions onto a Mass Flow Graph (MFG), allowing a pathway-based interpretation of metabolic flux distributions, and (iii) it applies a minimum-cut algorithm to extract critical pathways and compute Coefficients of Importance, which serve as pathway-specific weights in optimization. This framework ensures that metabolic flux predictions align with experimental data while maintaining a systematic understanding of how different pathways contribute to cellular adaptation.

### 2.2. Technical implementation

The **TIObjFind** framework was implemented in MATLAB, with custom code for the main analysis and the minimum cut set calculations performed using MATLAB’s maxflow package [30–32]. The minimum-cut problem can be solved using a variety of algorithms, including the Ford-Fulkerson, Edmonds-Karp [33], and Push-Relabel methods [34]. This study employed the Boykov-Kolmogorov algorithm due to its superior computational efficiency. It delivers near-linear performance across various graph sizes, significantly surpassing the performance of conventional algorithms [35,36]. Visualization of the results was done using Python, with the pySankey package. The scripts are available at the URLs listed in the Data Availability section.

### 2.3. Illustrating example

**Fig 1:**
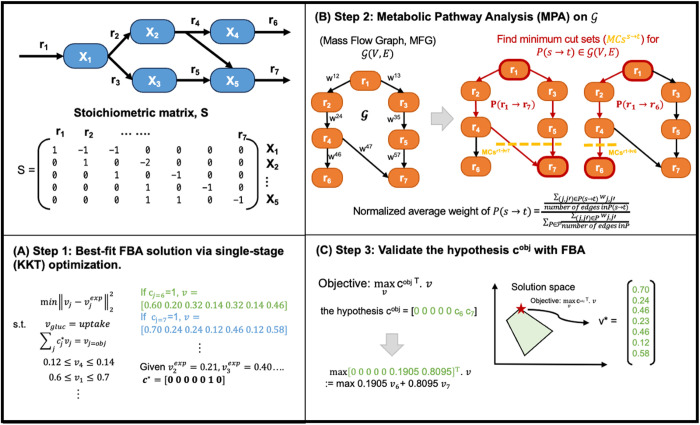
TIObjFind utilizes MPA to identify weighting coefficients, termed Coefficients of Importance, on reaction (r) within a metabolic network (metabolites *X*). (A) The framework consists of three main steps: Step 1: Identify a best-fit flux distribution using a single-stage (KKT) formulation of FBA, where candidate objectives *c* are tested against experimental fluxes ($v^{exp}$). The best-fit coefficient vector $c^{*}$ results a feasible flux solution v for each reaction r. **(B)** Step 2: Generate the Mass Flow Graph, G(V,E), based on FBA solutions and apply MPA. The Mass Flow Graph is a directed, weighted graph where edge weights *w* represent reaction fluxes. minimum cut sets (MCs) are identified using min-cut analysis between nodes, representing the minimal reaction sets needed to sustain flow from source to target (e.g., ${MCs}^{r_{\text{1}}\to r_{\text{7}}}$). The hypothesis coefficients $c^{obj}$ are calculated as the normalized average weights of the P(s→t). **(C)** Step 3: Re-apply FBA using the hypothesis coefficients $c^{obj}$ as the weighted combination of the target reactions. The updated FBA solutions incorporate these coefficients, providing an optimized flux distribution that aligns with the essential pathways identified by the MPA.

To illustrate the framework of **TIObjFind**, consider the toy model [37] shown in Fig 1. The model includes seven reactions and five metabolites. Coefficients of Importance are denoted as the coefficients $c_{j}^{obj}$ and $c^{obj}\;\cdot v$ serves as the weighted combination of fluxes (v) as the fitness function. Maximization of the distributed intracellular flux explains the experimental flux data. The framework is as follows: **(i) Step 1, Find best-fit FBA solutions using single-stage optimization**: Candidate objectives *c* are evaluated using a single-stage (Karush-Kuhn-Tucker, KKT) formulation of FBA that minimizes the squared error between predicted fluxes and experimental data ($v^{exp}$). For example, in the toy model, the objective is assigned to reaction *r*_6_ corresponding to c=[0,0,0,0,0,1,0]. This results a feasible flux distribution: $v_{j}^{*}=[\text{0}.\text{6}\text{0},\text{0}.\text{2}\text{0},\text{0}.\text{3}\text{2},\text{0}.\text{1}\text{4},\text{0}.\text{3}\text{2},\text{0}.\text{1}\text{4},\text{0}.\text{4}\text{6}]$. Using the derived solution, the metabolic fluxes between reactions can be represented as a directed, weighted graph referred to the Mass Flow Graph, G(V,E). **(ii) Step 2, Generate the graph and apply MPA**: Subsequently, Metabolic Pathway Analysis is applied to identify pathways that are essential for desired product formation (e.g., *r*_6_ and *r*_7_ are the extracellular product formation). MPA evaluates the contribution of each pathway to the overall flux distribution and examines the extent to which external metabolites taken up by the cell influence the pathway output. Here, minimum cut sets (MCs) are applied to identify essential pathways, represented as P(s →t), where *s* (e.g., r_1_) may refer to glucose uptakes, and *t* may represent *r*_6_ or *r*_7_, respectively. MCs are the minimal set of high-flux reactions necessary to sustain a flow from source to sink; their removal results in zero flux for the pathway (P(s→t)) at a steady state [28]. **(iii) Step 3, Identify Coefficients of Importance as the weights for objective function:** In G, the weights represent the probabilities that a me*t*abolite is produced or consumed by the source or target reaction, as defined in equation 4 (in Method section). The Coefficients of Importance (CoIs) for each target reaction are calculated as the normalized average of the weights of P(s→t), such that $\text{CoIs}\left(r_{\text{6}}\right)=\text{0}.\text{1}\text{9}\text{0}\text{5}$ and $\text{CoIs}\left(r_{\text{7}}\right)=\text{0}.\text{8}\text{0}\text{9}\text{5}$, ensuring that the sum of the CoIs is equal to one. These Coefficients of Importance serve as hypothesized coefficients for the distribution of fluxes and provide insight into which reactions or pathways are most critical under the experimental conditions.

To demonstrate the application of **TIObjFind**, two different systems were considered: (i) fermentation of glucose by *Clostridium acetobutylicum* (***Cac***); and (ii) the solventogenesis of the syntrophic consortium of engineered ***Cac*** and *Clostridium ljungdahlii* (***Clj***) for isopropanol, butanol, and ethanol production from glucose and carbon dioxide [38].

### 2.4. Determination of weighting factors for metabolic flux analysis based on normalized weights of the minimal pathways: Fermentation of glucose by *C. acetobutylicum*

#### 2.4.1. Stoichiometric model of solventogenic *C. acetobutylicum.*

The metabolic behavior of ***Cac*** is influenced by the balance between acidogenesis and solventogenesis [39,40]. During the acidogenic phase, the bacterium primarily converts glucose into organic acids such as butyrate and acetate, which leads to acid accumulation and a corresponding decrease in pH [39]. In contrast, when solventogenesis is activated, typically at a lower pH range of approximately 4.5 to 5.0, the metabolic flux shifts toward the production of solvents such as acetone and butanol [39,41]. This shift is essential in fermentation processes that aim to maximize solvent yield.

**Fig 2:**
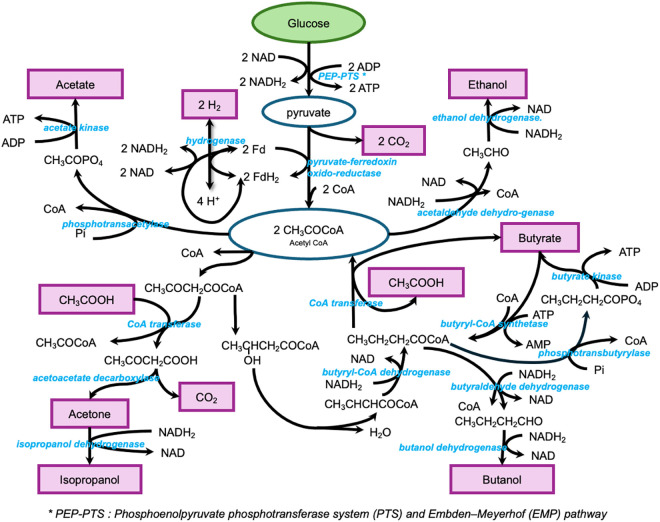
Metabolic pathway of glucose fermentation by *Cac* (based on Papoutsakis [43]). The key extracellular metabolites, highlighted in pink.

In this case study, two sets of experimental data from glucose batch fermentation by ***Cac*** were analyzed (Table 1) [42,43]. In Experiment 1, butanol production reached 28.9 mol per 100 mol of glucose fermented, whereas in Experiment 2, the yield was lower at 10.4 mol per 100 mol of glucose fermented. This difference correlated with butyrate accumulation, which was 36.4 mol per 100 mol glucose fermented in Experiment 1 and 54 mol per 100 mol glucose fermented in Experiment 2. To model the metabolic behavior of butyric acid bacteria, previous study developed a stoichiometric model that describes key biochemical transformations through lumped reactions [43]. These reactions summarize the overall metabolic activity of the organism (Fig 2). The metabolites highlighted in the purple box represent the main extracellular products produced by the bacteria. The stoichiometric model includes 14 metabolic species and 11 pathway fluxes (see S1 Text for more details).

**Table 1:** Comparison of experimental and calculated metabolite yields in *C. acetobutylicum* glucose fermentation.

	Exp1	Calculated				Exp2	Calculated			
	mol per 100 mol of glucose fermented									
Meta-bolite	Experi-mental	Eq. 10, No Weight	Eq. 10, Normalized Experi-mental Value as Weight	Eq. 10, TIObjFind-derived Weight	ObjFind	Experi-mental	Eq. 10, No Weight	Eq. 10, Normalized Experi-mental Value as Weight	Eq. 10, TIObjFind-derived Weight	ObjFind
**Butanol**	28.90	28.55	28.79	28.90	28.9	10.40	11.24	10.41	10.46	10.41
**Ethanol**	14.60	14.42	14.59	14.60	14.6	13.60	14.02	13.61	13.62	13.61
**H**_**2**_	169.00	168.93	169.95	169.00	175.85	186.00	186.26	186.95	186.73	186.95
**Acetone**	10.00	9.41	9.97	9.99	10	0.00	2.14	3.11	3.32	3.11
**Acetoin**	5.40	5.18	5.39	5.32	5.4	5.10	5.41	5.10	5.10	5.10
**CO**_**2**_	206.00	206.50	208.71	206.48	214.15	194.00	192.85	192.64	193.72	192.64
**Acetate**	24.10	24.06	24.02	23.90	24.1	30.70	30.59	30.68	30.51	30.68
**Butyrate**	36.40	36.18	36.14	35.33	36.4	54.00	54.31	54.03	54.30	54.03
**IPA**	0.00	0.00	0.00	0.00	1.35	0.00	0.00	0.00	0.00	0.00

However, the degree of redundancy in the stoichiometric matrix can lead to the introduction of a singularity. One way to address this issue is by measuring one of the fluxes in the singular group. By fixing one of these fluxes with additional measurements, the remaining fluxes in the singular group can be calculated using the stoichiometric matrix [43]. Additionally, redundant systems can be solved using a least-squares approach [40]. The least-squares solution is the set of fluxes that minimizes the weighted sum of squared differences between the observed values and the calculated values.

#### 2.4.2. Minimization of weighted squared residuals in flux estimation.

The basis of metabolic flux analysis has been thoroughly outlined in previous study. A concise overview is provided here for context. Following the method described by Desai (1999), the stoichiometric model for solventogenic *Clostridia* can be formulated as a constrained minimization problem, expressed as [40]:


$${min\;\left\|W^{-\text{1}}S\cdot v-W^{-\text{1}}b\right\|}_{\text{2}}^{\text{2}}$$ (1)

The least-squares solution (v) to the problem is the set of fluxes that minimizes the sum of weighted squared residuals between observed values (second term of equation 1) and calculated values (first term of equation 1). where *S* is the stoichiometric matrix representing reaction coefficients; *b* represents the accumulation vector for metabolites within the network; and *W* contains weighting factors that account for the relative importance of measured accumulation terms. Unlike the standard pseudo-steady-state assumption, which sets the accumulation terms to zero (e.g., equation 8: $\sum_{j=\text{1}}^{M}S_{ij}v_{j}=\text{0},\;\forall i\in N$), this formulation explicitly considers metabolite accumulation, particularly for exchangeable species (e.g., fermentation products: isopropanol, ethanol, etc.) that are transported across the cell membrane. The accumulation terms for exchangeable species must be measured directly, if not, the associated balance in the stoichiometric matrix should be removed.

#### 2.4.3. Determination of the weighting factors.

Weighting factors (*W*) are used to prioritize specific measurements based on their biological significance. As suggested by Desai et al., the weighting factor for components such as glucose, biomass, and acetate is determined by the standard deviation of each measurement [40]. In the case of metabolic intermediates, the weighting factor is based on the largest flux magnitude among the pathways in which the intermediate is involved [40]. The scaling factor affects the degree to which the pseudo-steady-state approximation is enforced.

In this study, three different weighting approaches are analyzed. (i) No weighting factors: In this case, all elements of *W* are set to one, making equation 1 equivalent to ${min\;\left\|S\cdot v-b\right\|}_{\text{2}}^{\text{2}}$, treating all measurements equally without prioritization. (ii) Using normalized experimental values as weights: Here, experimental values for accumulation components, including glucose, butyrate, butanol, acetone, ethanol, acetoin, and isopropanol, are used to determine reaction fluxes. The unmeasured metabolites are estimated based on mass balance constraints in the stoichiometric matrix. The calculated metabolite flux values are then normalized by the total sum of metabolites fluxes, ensuring that all values remain within the range of 0–1. A larger weight indicates a higher flux magnitude toward product synthesis, which reduces its influence on enforcing the pseudo-steady-state approximation, whereas lower flux magnitudes impose a greater effect. (iii) Using **TIObjFind**-derived weights: In this case, the measured accumulation of key components (glucose, butyrate, butanol, acetone, ethanol, acetoin, and isopropanol) is fixed. Reaction fluxes are then computed based on mass balance, resulting in eleven predicted fluxes. The sum of pathway weights is back-calculated with the predicted fluxes using the **TIObjFind** framework, and the normalized weights of the minimal pathways serve as weighting factors in equation 1. (iv) the predicted fluxes using ObjFind method with the set of possible *c*_*j*_ values consistent with the minimization of the sum-squared difference between a subset of observed fluxes ($v_{exp}\text{:}$ isopropanol dehydrogenase, butyraldehyde dehydrogenase, butanol dehydrogenase, butyrate kinase, butyryl-CoA synthetase, ethanol dehydrogenase, and acetaldehyde dehydrogenase, acetate kinase)

For both weighting cases (ii) and (iii), scaling factors for the cofactors and metabolic intermediates are applied as suggested by previous work [40]. Specifically, a scaling factor of 0.01 is assigned for NADH₂ and FdH₂, while a scaling factor of 0.1 is used for pyruvate and acetyl-CoA [40]. Additionally, if the accumulated metabolite concentration is zero, the weighting factor is set to one to maintain consistency within the metabolic network.

#### 2.4.4. Validation of the weighting factors from Coefficients of Importance.

**Fig 3:**
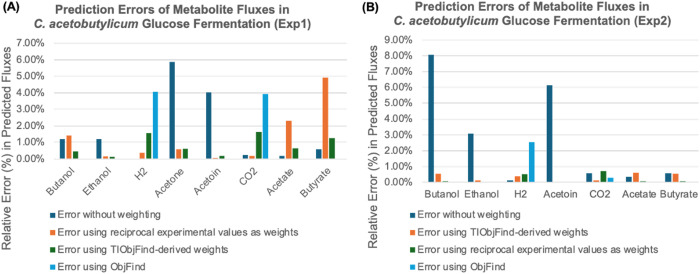
Comparison of prediction errors in metabolite fluxes for *C. acetobutylicum* glucose fermentation in Experiment. **(A)** Experiment 1, **(B)** Experiment 2. Three different weighting strategies were applied for flux prediction: **(i)** No weighting, **(ii)** Normalized experimental values as weights, **(iii) TIObjFind**-derived weights, and **(iv)** ObjFind method. Comparison of relative errors (%) in predicted fluxes for different weighting methods. Errors are calculated as $\left|\frac{Predicted-Experimental}{Experimental}\right|\times \text{1}\text{0}\text{0}\%$.

Fermentation of glucose by ***Cac*** was analyzed using experimental data from van der Lek [42] reported in paper [43], with metabolite yields reported in moles per 100 moles of glucose fermented. The calculated values were obtained using equation 10 under three different weighting strategies: no weighting, normalized experimental values as weights, and **TIObjFind**-derived weights. Table 1 and Fig 3 compare the experimental data with the predicted values obtained from these approaches.

The results indicate that applying **TIObjFind**-derived weights and normalized experimental value weights significantly reduces prediction errors for most metabolites compared to unweighted calculations (Fig 3). Both weighting approaches (ii) and (iii) show similar performance, with only minor deviations in specific metabolites. Notably, acetate and butyrate predictions using **TIObjFind**-derived weights exhibit slightly higher errors than those obtained using normalized experimental values, though the relative error remains below 3%. This finding demonstrates that end reactions tend to closely match experimental data, while acetate and butyrate function as intermediates, participating in both production and uptake for other end products (see S2 Text for additional discussion). Also, it is important to note that the relative error in predicted values depends not only on experimental error but also on the magnitude of the predicted value itself.

On the other hand, when applying the ObjFind framework, the prediction error for most measured metabolites was close to zero, but the predictions for H₂ and CO₂ were relatively poor. In practice, the use of ObjFind in this case depends on intracellular fluxes (e.g., isopropanol dehydrogenase, butyraldehyde dehydrogenase) that are typically estimated using isotope-labeling experiments such as ^13^C metabolic flux analysis. Providing these internal fluxes as constraints allows ObjFind to distribute the coefficients of importance (*c*_*j*_) across multiple reactions/ or pathways and achieve biologically realistic solutions. While ObjFind is flexible in either extracellular or intracellular fluxes, the lack of intracellular measurements less constrained the feasible solution space. This led to accurate prediction of the measured metabolites, but relatively poor predictions for unmeasured metabolites (e.g., H₂, CO₂). In contrast, the proposed TIObjFind framework recalculates pathway-level weights from the best-fit FBA solution, enabling its application even under data-limited conditions.

Overall, these results demonstrate that TIObjFind provides reasonable predictions and, in this case study, achieves accuracy within a similar level of acceptance as normalized experimental weights. The main strength of TIObjFind, however, lies in its ability to incorporate metabolic pathway analysis and assign Coefficients of Importance at the pathway level. Because Case Study 1 is based on a lumped stoichiometric network, this advantage is less apparent here. To illustrate the unique value of TIObjFind, an additional E. coli case study is presented in the S2 Text, where pathway-level differences under varying environmental conditions become evident.

### 2.5. Identifying the objective function that best aligns with experimental flux data in an anaerobic co-culture system

#### 2.5.1. Description of the co-culture system of *C. ljungdahlii* and *C. acetobutylicum.*

The second case considered here was investigated by Charubin and Papoutsakis [38]. Studies have shown that when grown in syntrophic coculture, these species can fuse membranes and exchange cytosolic contents, resulting in the formation of hybrid cells [32,44]. These hybrid cells exhibit significant shifts in gene expression and altered growth phenotypes, distinguishing them from nonhybrid cells.

The initial conditions for the batch culture included a mixture of 10 mL fructose (from the 100X Solution (500 g/L)) and 442 mM glucose, supplemented with Turbo Clostridium Growth Medium (CGM), featuring a diverse range of nutrients and mineral salts. To train and validate the growth kinetic model, coculture samples were collected approximately every 10 hours until the 50-hour mark, corresponding to the late stationary phase of the culture. The specific growth rate of each organism was estimated using optical density measurements at a wavelength of 600 nm (OD600). The relative abundance of each species, ***Cac*** and ***Clj***, in the coculture was ascertained through quantitative PCR (qPCR) for genome copy numbers. Flow cytometry and fluorescence microscopy were employed to determine the relative abundance of hybrid cells, nonhybrid ***Cac*** cells, and nonhybrid ***Clj*** cells. Metabolic model comparison and analysis utilized measurements of extracellular glucose, fructose, lactate, acetate, butanol, ethanol, and isopropanol titers, gathered using high-pressure liquid chromatography. The experimental data utilized in this study can be found in S1 Data.

**Fig 4:**
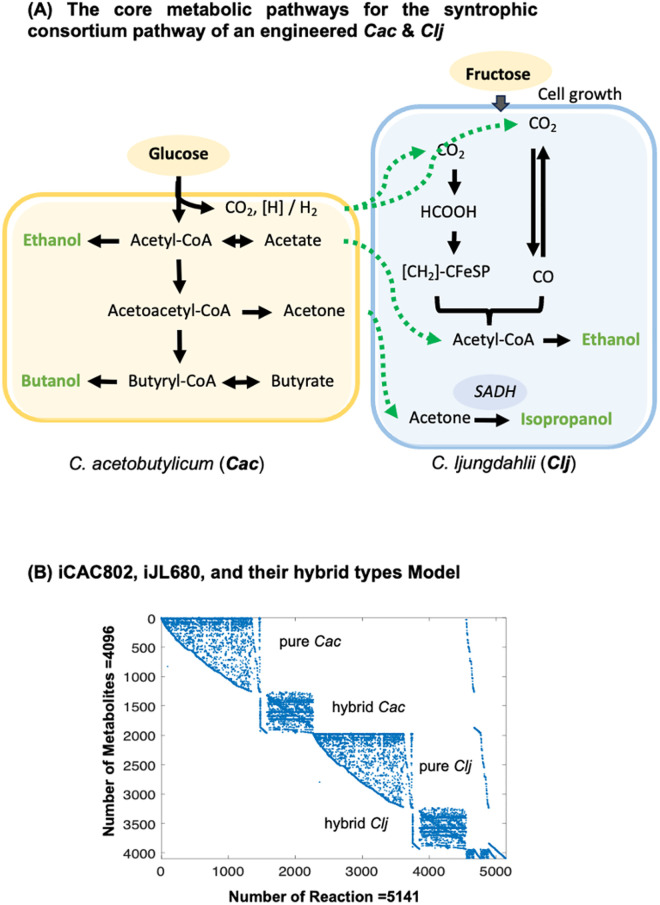
A graphical representation of the syntrophic consortium model. (A) The core metabolic pathways for the syntrophic consortium pathway of an engineered ***Cac*** & ***Clj*** to produce isopropanol, butanol, and ethanol from glucose and carbon dioxide. **(B)** Microbial metabolic networks spy plot of the iCAC802, iJL680, and their hybrid types of models. The dots represent that the corresponding metabolites are associated with the reactions.

The core metabolic reactions of the co-culture system involve ***Clj*** converting the acetone, (***Cac***, into isopropanol (IPA) [38]. Additionally, ***Clj*** employs the Wood-Ljungdahl pathway to utilize CO_2_ and H_2_, resulting from the production of acetate and a portion of ethanol [38] (Fig 4A). The genome-scale model describing the IBE mix-alcohols fermentation process involves multiple species, including ***Cac***, ***Clj***, and hybrid types of ***Cac*** and ***Clj***. The metabolic network for nonhybrid and hybrid cells was based on the models including iCAC802, iJL680, and the flux variability analysis with hybrid and nonhybrid models from Foster C. et al., work [32]. The resulting stoichiometric matrix has size 4096×5141 (Fig 4B). Alongside this, experimentally measured fermentation data points for the solventogenesis [38], including ethanol, isopropanol, butanol, and acetate (an indicator of metabolic state) were used to compare the predicted and experimental extracellular fermentation product titers. This case study identifies the Coefficients of Importance for the objective function that best aligns with experimental flux data for complex metabolic systems.

#### 2.5.2. Data preparation for the TIObjFind framework.

To address the complexity of dynamic multispecies performance, a **TIObjFind** framework was implemented within the dynamic multispecies metabolic modeling framework (DMMM) [32]. This framework integrates genome-scale dFBA and an ordinary differential equation-based growth model, encompassing non-hybrid and hybrid cell states. It is important to note that the DMMM framework is a two-stage single objective procedure, in which the first stage is to maximize cells’ growth, and the second stage is to optimize the specific solvent corresponding to the solventogenic shifts. In this work following the DMMM framework, the hypothesis coefficients of the second-stage optimization objective function were determined by the Coefficients of Importance via **TIObjFind**.

First, following the steps of the framework, a Mass Flow Graph and pathways reflecting the metabolic blueprint were constructed. These minimal pathways encompass the relevant nodes (reactions) and weights (flux probabilities), focusing on the specified source (*s*) and sink (t) reactions. The source metabolites include glucose and fructose, while the sink metabolites consist of ethanol, isopropanol, butanol, and acetate (based on the main products described in Fig 4B). For fructose as the source, four minimal pathways were identified for each sink metabolite and species, resulting in a total of 16 minimal pathways (4 species: nonhybrid ***Cac***, hybrid ***Cac***, nonhybrid ***Clj*** and hybrid ***Clj***; with 4 sink metabolites). For glucose as the source, nonhybrid ***Clj*** can only uptake fructose, leading to the determination of 12 minimal pathways (3 species: nonhybrid ***Cac***, hybrid ***Cac***, and hybrid ***Clj***; with 4 sink metabolites).

Second, normalize of the weights, $w_{j,j^{\prime }}$. Upon identifying the 28 minimal pathways (Table 2) and their corresponding total sum of weights, the sum of weights of the four sink metabolites (t) was normalized to ensure all features and variables remained on a consistent scale. The normalized weight of the minimal pathway targeting t, referred to as CoIs(t), can then be incorporated into equation (7) alongside the four objectives (the normalized weights for product secretion at each time point see Supplementary Information S1 Data).

**Table 2:** 28 combinations of source (s) and a sink (t) for each species. Non-hybrid *Cac* was engineered to be an IBE producer. In contrast, non-hybrid *Clj* can only use fructose.

s\t	Glucose	Fructose
Butanol	Nonhybrid ***Cac***/ hybrid ***Cac***/ hybrid ***Clj***	Nonhybrid ***Cac***/ hybrid ***Cac***/ Nonhybrid ***Clj***/ hybrid ***Clj***
IPA	Nonhybrid ***Cac***/ hybrid ***Cac***/ hybrid ***Clj***	Nonhybrid ***Cac***/ hybrid ***Cac***/ Nonhybrid ***Clj***/ hybrid ***Clj***
Acetate	Nonhybrid ***Cac***/ hybrid ***Cac***/ hybrid ***Clj***	Nonhybrid ***Cac***/ hybrid ***Cac***/ Nonhybrid ***Clj***/ hybrid ***Clj***
Ethanol	Nonhybrid ***Cac***/ hybrid ***Cac***/ hybrid ***Clj***	Nonhybrid ***Cac***/ hybrid ***Cac***/ Nonhybrid ***Clj***/ hybrid ***Clj***

**Fig 5:**
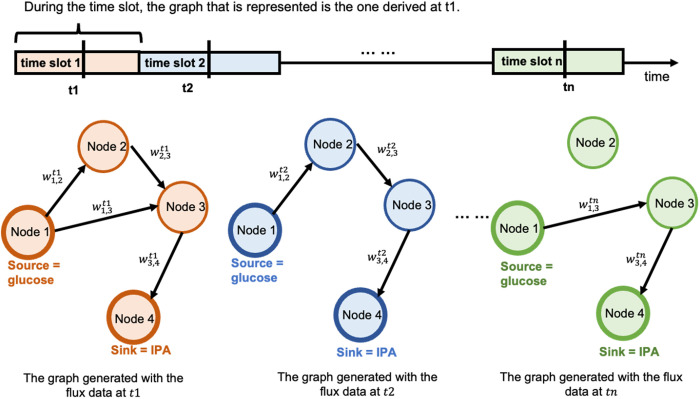
Graphical representation of the implementation of the TIObjFind with experimental data.

Lastly, update the Coefficients of Importance for the objective function over time. In this study, the hypothesis coefficients of the objective function, as defined in equation (6), are updated at each time point within the specified time interval. As an example, Fig 5 illustrates a minimal pathway for *t*1, identified using the minimum cut sets (MCs), with a set of weights ($w_{\text{1},\text{2}}^{t\text{1}},\;w_{\text{2},\text{3}}^{t\text{1}},\;w_{\text{1},\text{3}}^{t\text{1}},\;w_{\text{3},\text{4}}^{t\text{1}}$), depicting the intercellular reaction relationships for time slot 1. To account for environmental changes over time, the minimal pathway evolves into another one generated using flux data from *t*2, with a set of weights of ($w_{\text{1},\text{2}}^{t\text{2}},\;w_{\text{2},\text{3}}^{t\text{2}},\;w_{\text{3},\text{4}}^{t\text{2}}$), and so on. The hypo*t*hesis coefficients of the objective function remain consistent across time slots. The source (e.g., glucose uptake) and sink reactions (e.g., IPA formation) are provided in Table 2. This method offers the advantage of minimal data requirements and reduced computational burden.

#### 2.5.3. Identify the objective function with Coefficients of Importance that best aligns with experimental flux data.

A flux distribution for the central metabolic network was characterized experimentally then serves as constrains in FBA. The desired products titers distribution was obtained by HPLC and corresponds to bacteria cultivated in batch culture with glucose and fructose as the limiting substrate. Table 2. shows the weighted reaction for the desired products’ production for which the metabolic network is optimized. The hypothesis Coefficients of Importance for the target reactions: Butanol, Isopropanol, Acetate, and Ethanol, were evaluated across multiple time points to observe changes in metabolic priorities during different fermentation phases: Acidogenic, 1^st^ Solventogenesis, and 2^nd^ Solventogenesis.

During the initial hours of fermentation, the co-culture demonstrated a preference for acetate production pathways, as indicated by the high Coefficients of Importance for Ac (0.63 at t = 2.5 hr and 0.18 at t = 11.9 hr). This result suggests that acetate production was favored as a metabolic overflow pathway, also enabling the cell to grow more rapidly and achieve higher cell densities [45]. During acidogenesis, ***Cac*** primarily produces acetate, which is the main product of this phase. As the culture transitioned from the Acidogenic to the Solventogenesis phase around t = 20 hr, the Coefficients of Importance shifted, reflecting a change in metabolic focus. As Table 3 shown, the Coefficients of Importance for isopropanol increased significantly to 0.25, indicating an increased priority for IPA production. Production of butanol and acetate remained active, with Coefficients of Importance of 0.24 and 0.28, respectively, demonstrating that multiple solvents and byproducts continued to be produced. In the later time points of the 2^nd^ Solventogenesis phase, particularly at t = 32.3 hr and afterward, the Coefficients of Importance reveal a pronounced shift toward IPA production, with the weight for IPA reaching 0.56. Butanol and ethanol maintained moderate Coefficients of Importance, indicating continued but less prioritized production, while acetate’s weight dropped to 0.12, as acetic acid was taken up during the solvent production phase and activated to its respective CoA thiolesters.

**Table 3:** The hypothesis coefficients $c^{obj}$ as the weighted combination of the target reactions.

Objective	t = 2.5 hr	t = 11.9 hr	t = 19.8 hr	t = 32.3 hr
**Butanol**	0.14	0.24	0.24	0.11
**Isopropanol**	0.15	0.26	0.25	0.56
**Acetate**	0.63	0.18	0.28	0.12
**Ethanol**	0.08	0.31	0.23	0.21

#### 2.5.4. Validation of Coefficient of Importance with experimental data.

Experimental fermentation profiles highlight two notable events within this system. The first is observed around the 12-hour mark when the exponential accumulation of acetate stops, and the exponential solventogenesis of IPA, ethanol, and butanol begins. The second significant metabolic shift is observed around the 30-hour point, during which the accumulated acetate in the medium starts to be consumed.

**Fig 6:**
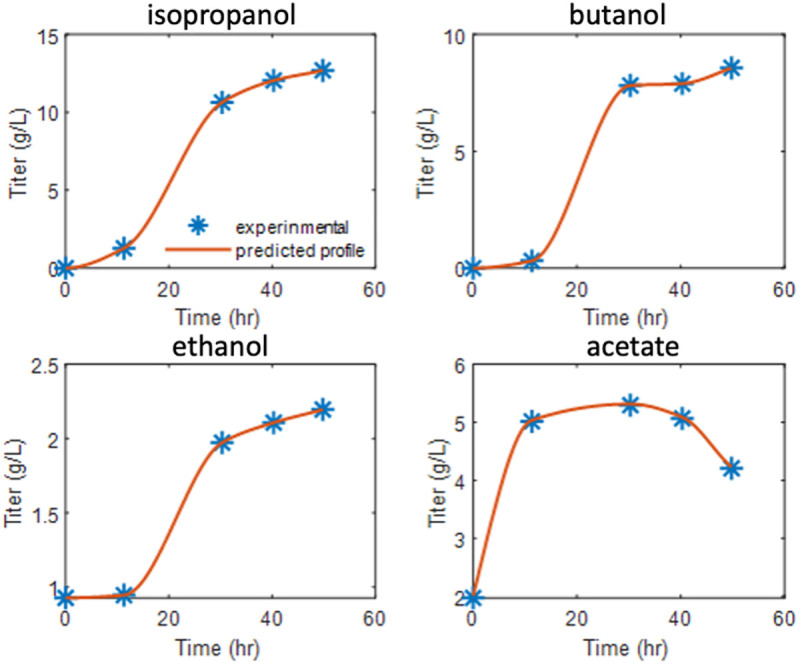
Predicted vs. experimental titers using graph-based multi-objective FBA in DMMM.

As shown in Fig 6, the predicted flux distributions have displayed agreement with experimentally with the use of the Coefficients of Importance-based weighting in objective function in the second stage of DMMM framework optimization. The match between **TIObjFind**-derived predictions and experimental fluxes suggests that the framework identifies the best-fit objective function for metabolic modeling.

**Fig 7:**
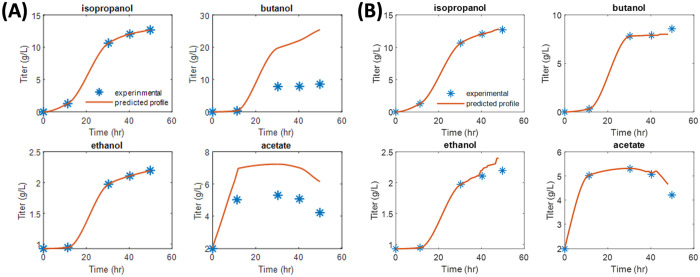
Predicted and experimental fermentation titers under single-objective function in the second stage of DMMM framework. **(A)** The objective for the first 12 hr is butyrate and after that time is butanol. **(B)** The objective for the first 12 hr is butyrate and after that time is L-lactate.

In contrast, the use of single-objective FBA in the second stage of DMMM framework optimization treats the secretion products as potential objectives among thousands of reactions. The manual adjustment of constraints is necessary to account for metabolic shifts, particularly when the culture transitions between consuming and producing fermentation products. Therefore, we followed the same objectives used in the DMMM model by Foster et al. [32], which employed butyrate, butanol, and ethanol as objective functions in the second-layer optimization. Despite using the same objectives, the model’s predictions did not align well with experimental data, as shown in Fig 7A. In particular, butyrate was the sole optimization objective for the first 12 hours, after which the optimization focus shifted exclusively to butanol. The predicted flux distributions in Fig 7A closely match the titers of isopropanol and ethanol but fail to accurately capture butanol and acetate profiles beyond the 12-hour mark.

To further evaluate our framework against state-of-the-art methods, we applied the “step 1” solely in the **TIObjFind** framework, which corresponds to the **ObjFind** method [22]. This step identifies the objective by minimizing the sum of squared deviations from experimental data. In this analysis, butyrate was determined to be the optimal objective function up to 12 hours, whereas L-lactate, a product of anaerobic metabolism, became the primary objective at later time points. The predicted flux distributions using the **ObjFind** method with FBA are presented in Fig 7B. While the method captures profiles before the 40 hour, butanol, ethanol, and acetate predictions did not fit well at the final stages of the reaction profile. The SSE values for the **ObjFind**-derived objective function increased after 40 hours, specifically, the SSE values for isopropanol, acetate, butanol, and ethanol titer were 0.09, 0.09, 0.32, and 0.06, respectively. While the SSE values for the **TIObjFind**-derived objective function were approximately 0, rounded to four decimal places.

The comparison indicates that even when using the same objective function as in previous modeling, the different environmental conditions of the system can lead to the objective function being unsuitable for the current case, despite the co-culture being the same. On the other hand, the objective function derived from **ObjFind**, which treats butyrate and lactose production as objectives in different phases, provides a satisfactory fit with the experimental results. Although it may seem non-intuitive to consider these reactions as objectives. In contrast, the **TIObjFind** determined Coefficients of Importance-based weights based on the minimal pathways from the Mass Flow Graph, with the weights summing up to 1, allowing every component to contribute to the objective function. The high Coefficients of Importance reveal the reactions or pathways that are most important under the experimental conditions. By adjusting these Coefficients of Importance, **TIObjFind** can tune the objective function to more accurately align with observed experimental data.

### 2.6. Computational cost

All computations were performed on a desktop workstation equipped with an Intel Core i7-11850H processor (2.50 GHz) and 64 GB RAM. For the reduced stoichiometric network in Case Study 1, a single TIObjFind run completed within a few seconds. For the genome-scale model in Case Study 2, the runtime increased to approximately 10 seconds per optimization. The complete workflow for Case Study 2, which included multiple time points, repeated optimization runs, and minimum cut set enumeration, required ~100 minutes in total (6,598.77 s). This runtime includes 480 individual FBA evaluations, each solving within 8–15 s. The computational cost in TIObjFind arises primarily from repeated dual optimization and cut-set calculations.

## 3. Conclusion

In this work, we present a novel topology framework named Topology-Informed Objective Find (**TIObjFind**) that imposes metabolic pathway analysis with network stoichiometry and experimental flux data to determine the most likely objective function for a given biological system. **TIObjFind** enhances **ObjFind** by integrating predefined FBA-derived flux distributions with MPA. Using minimal pathway analysis within the Mass Flow Graph, **TIObjFind** determines Coefficients of Importance that quantify each reaction’s contribution to a predefined cellular objective. This framework relies on FBA as a foundation, applying Coefficients of Importance-based weighting to align the model with experimental flux data. By identifying minimum cut sets in the Mass Flow Graph, **TIObjFind** isolates essential pathways, offering insights into metabolic flexibility and adaptation. This dual-layered approach provides a more comprehensive view of metabolic changes, particularly under varying environmental conditions, revealing shifts in pathway utilization while grounded in the FBA-derived objectives.

The framework applied to two case studies: fermentation of glucose by ***Cac*** and a co-culture solventogenesis system, demonstrates its utility in capturing dynamic metabolic adaptations. The framework aligns predicted flux distributions well with experimental data, showing the ability in identifying key metabolic shifts in both single-species and multi-species systems. In the second case study, the titer values of isopropanol, acetate, butanol, and ethanol were assessed using Coefficients of Importance-based weighting objective functions. The SSE values of the weighting-objective function outperformed the single-objective function, indicating that it was more effective.

Despite these advances, certain challenges remain. Unlike methods like FlexFlux, which extends FBA by integrating qualitative gene regulatory networks to constrain flux distributions, TIObjFind does not require predefined regulatory logic. Instead, **TIObjFind** relies on experimental flux data and comprehensive network models to ensure accuracy. As network size and objective complexity increase, computational demands increase due to the repeated procedure of minimal-cut calculations required to determine pathway weights of different target reactions. Scaling up **TIObjFind** to genome-scale models will necessitate optimizing computational efficiency. Furthermore, the limited availability of large-scale, experimentally-derived flux datasets poses an ongoing hurdle, as flux data are essential for validating **TIObjFind**’s predictive capabilities.

## 4. Method

The following sections describe the graph-based representation of flux solutions, the computation of Coefficients of Importance using the minimum-cut algorithm, and the application of **TIObjFind** to infer the Coefficients of Importance of metabolic objectives. A list of mathematical notations is provided in S3 Text.

### 4.1. Mapping the flux balance analysis solutions onto the mass flow graph

In this work, the metabolic networks were represented as reaction adjacency graphs [26], where reactions are represented as nodes. Nodes are connected if the reactions share metabolites, with compound information stored as edge labels. The graphs can be derived from the stoichiometric matrix (*S*), calculated through the multiplication of two Boolean stoichiometric matrices described as: ${Boolean(S)}^{T}\;\cdot Boolean(S)$. Building on the graph structure, the active or inactive metabolic reactions under different environmental conditions are represented by edge weights, which are derived from the probabilities that a metabolite is produced or consumed by an upstream or downstream reaction. This representation is referred to as the Mass Flow Graph.

Mass Flow Graph (MFG) is a flux-based weighted graph proposed by Beguerisse-Diaz et al [26]. The following outlines the process of constructing a flux-based weighted graph for metabolic networks [26,46], which is represented by an n × m stoichiometric matrix, *S* (metabolite i = 1, …,n; reaction j = 1, …,m). First, the graph’s structure should be constructed, including nodes and edges incorporating the irreversibility of reactions. To account for both forward and reverse fluxes in the graph, the stoichiometric matrix must be unfolded with reaction reversibility information. Thus, a diagonal matrix, diag(rev), is used where *rev* being an m-dimensional vector with ${rev}_{j}=\text{1}$ denoting the reversible reactions and ${rev}_{j}=\text{0}$ the irreversible ones. The unfolded version of the stoichiometric matrix, $S_{\text{2}m}$ represents the 2*m* forward and reverse reactions and is expressed in equation (2) [26]:


$$\begin{array}{c} S_{\text{2}m}=\;[S\;-S][\begin{array}{cc} I_{m\times m} & \text{0}\\ \text{0} & diag(rev) \end{array}] \end{array}$$ (2)

The $S_{\text{2}m}$ structure matrix has dimensions n×2m. Following this, two matrices are derived from $S_{\text{2}m}$: the production stoichiometric matrix, $S_{\text{2}m+}$, and the consumption matrix, $S_{\text{2}m-}$. The production stoichiometric matrix, $S_{\text{2}m+}$, represents the quantities of metabolites produced by the reactions and can be defined as $\frac{\text{1}}{\text{2}}(abs\left(S_{\text{2}m}\right)+S_{\text{2}m})$. On the other hand, the consumption matrix, $S_{\text{2}m-}$, which signifies the quantities of metabolites consumed in the reactions, can be defined as $S_{\text{2}m-}=\frac{\text{1}}{\text{2}}(abs\left(S_{\text{2}m}\right)-S_{\text{2}m})$.

The weight of the edge between reactions *j* and $j^{\prime }$ is defined as the total mass flow of metabolites produced by *j* and consumed by $j^{\prime }$. To accomplish this, the FBA solution vector, v, is decomposed into forward and backward components as shown in equation (3) [26]:


$$v_{\text{2}m}=[\begin{array}{c} v_{+}\\ v_{-} \end{array}]=\frac{\text{1}}{\text{2}}[\begin{array}{c} abs(v)+v\\ abs(v)-v \end{array}]$$ (3)

where positive entries in the FBA solution correspond to forward fluxes ($v_{+}$), while negative entries correspond to backward fluxes ($v_{-}$). The weights for each edge in the flux-based weighted graph are determined by calculating the ratio of partial mass flows to the total mass flow, represented in equation (4) as an adjacency matrix [26]:


$$MFG={\left(S_{\text{2}m+}\cdot diag\left(v_{\text{2}m}\right)\right)}^{T}{diag(S_{\text{2}m+}\cdot \;v_{\text{2}m})}^{†}{(S}_{\text{2}m-}\cdot diag(v_{\text{2}m}))$$ (4)

where the superscript (†) denotes the Moore-Penrose pseudoinverse which can be applied to any matrix, including non-square and singular matrices [47]. The graph weights are determined by considering the consumption or production of metabolites based on their corresponding flux, thus satisfying the stoichiometric constraints. This approach ensures that the graph captures the flow dynamics of the metabolic network while taking into account the flux directionality and mass balance constraints.

### 4.2. Metabolic pathway analysis with the minimum-cut algorithm and Coefficients of Importance determination

The minimum-cut algorithm identifies the edges with the smallest weights, and their removal would result in disconnecting a specified source from its corresponding sink within a network [48]. This algorithm has been widely applied in various fields, such as network flow, transportation, and computational biology [49–51]. This algorithm provides valuable insights into the relative availability of metabolites within the overall metabolic network. minimum cut sets (MCs) are sets of reactions whose removal will disable the function of a given target reaction, t. They are typically related to failure modes of extreme pathways and elementary modes. Therefore, the unique decomposition of the network into pathways P(s→t) highlights potentially important modes.

The problem describes as follows: Given a directed graph G(V,E) with a source node *s*, a sink node *t*, and a set of edge capacities $C(j,j^{\prime })$ ≥ 0 for every $(j,j^{\prime })\in E$. The objective is *t*o find the cut set, ${MCs}^{s\to t}\subseteq E$, that partitions the graph into two disjoint sets S and *T*, where s∈S and t∈T, and the sum of the capacities of the edges crossing the cut is minimized. The minimal pathway, P(s→t), ensure flux or flow from *s* to *t*. The average weight of a pathway P(s→t) is given by:


$$\text{Average}\;\text{weight}\;\text{of}\;P(s\to t)=\;\frac{\sum_{\left(j,j^{\prime }\right)\in P(s\to t)}w_{j,j^{\prime }}\;}{number\;of\;edges\;in\;P(s\to t)}$$ (5)

where $w_{j,j^{\prime }}$ is the weight of edge $\left(j,j^{\prime }\right)$ in the pathway P(s→t). Let P be the set of all pathways on the graph from the defined sources to the targets. To reflect the interdependent distribution of control over the total network flux, the Coefficients of Importance (or CoIs and weights) of the desired products synthesis are normalized. The normalized average weight of the desired pathway is:


$$\text{CoIs}(\text{t})=\text{Normalized}\;\text{average}\;\text{weight}\;\text{of}\;\;P(s\to t)=\;\frac{\text{Average}\;\text{weight}\;\text{of}\;P(s\to t)\;}{\sum_{P\in P}\frac{\sum_{\left(j,j^{\prime }\right)\in P}w_{j,j^{\prime }}\;}{number\;of\;edges\;in\;P}}$$ (6)

The CoI(t) derived from the weights based on the mass flow of minimum number of reactions that exist as a functional unit (target reactions, t). Thus, higher Coefficients of Importance values indicate more active and essential pathways associated with the target reaction.

### 4.3. Hypothesized metabolic objective functions

FBA is a mathematical method in computational biology used to model metabolic fluxes in biological systems [52,53]. Linear equations are used to optimize reaction fluxes to achieve specific objectives (see S2 Text). When optimizing for multiple objectives, a common approach is to combine them into a single scalar fitness function using a weighted sum, where the weights represent the relative importance of each objective [54]. In our framework, the scalar fitness function is expressed as:


$$Z=\;c^{obj}\cdot v$$ (7)

where $c^{obj}$ is the vector of Coefficients of Importance, and v is the flux vector. The Coefficients of Importance are determined using MPA, specifically through the identification of Minimal Cut Sets in the Mass Flow Graph. The mathematical expression where the $c^{obj}\;\cdot v$ is not solely a reaction, but a combination of fluxes that corresponding to certain conditions, allows us to explore a range of possible solutions, rather than limiting the analysis to just one optimal solution.

**Fig 8:**
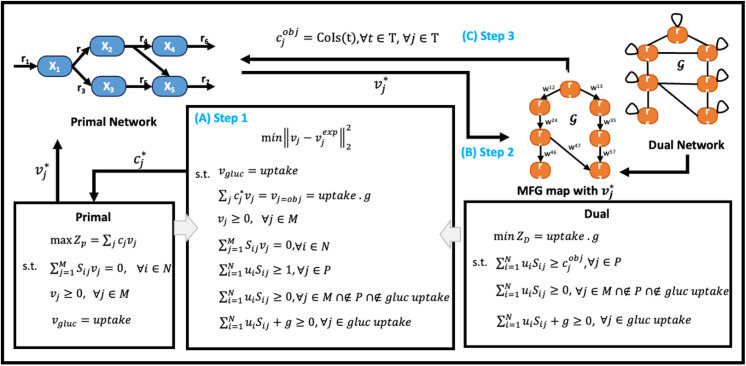
The optimization-based framework of TIObjFind. **(A)** Step 1 reformulates the optimization problem as a single-level problem using the duality theorem of linear programming, subject to thermodynamic, mass balance, and uptake constraints. These dual variables, *u*_*i*_ and *g*, reflect the sensitivity of the optimal objective value *Z*_*p*_ to changes in their associated constraints. The reaction fluxes $v_{j}$ are the dual variables for the dual constraints, and $c_{j}^{*}$ denotes weights for any potential cellular objective (e.g., biomass formation or energy production). The computed fluxes $v_{j}^{*}$ are then mapped in the dual network. (B) Step 2 maps the FBA solutions, $v_{j}^{*}$, onto the Mass Flow Graph. In the dual formulation, primal reactions become metabolites in the dual network, while primal metabolites serve as constraints in the dual. Self-loops represent autocatalytic reactions, where products also act as reactants, capturing internal metabolic fluxes. **(C)** Step 3 shows the normalization of the pathway importance (represented as edge weights, *w*, in the Mass Flow Graph), leading to a new objective reaction flux distribution.


In the **TIObjFind** framework, FBA is applied in two stages: (i) to identify the optimal objective reactions that minimize discrepancies with experimentally measured data, and (ii) to validate the Coefficients of Importance (CoI) by applying CoI-based weighting in the objective function to assess its alignment with experimental data. This approach enables **TIObjFind** to reverse-engineer and determine the cellular objective through an FBA-based methodology [55]. As shown in Fig 8, **TIObjFind** first solves a bi-level optimization problem, minimizing the sum of squared errors between experimental fluxes and the FBA-derived solution while maximizing a cellular objective:

$$min{\left\|v_{j}-v_{j}^{exp}\right\|}_{\text{2}}^{\text{2}}\;$$

$$\text{s}.\text{t}.\;\text{max}\;v_{j=obj}$$

$$s.t.\;\sum {\mathrm{\backslash nolimits}}_{j=1}^{M}S_{ij}v_{j}=0,\;\forall i\in N$$

$$v_{j}\geq \text{0},\;\forall j\in M$$

$$\;\;\;\;\;\;\;\;\;\;\;\;\;\;\;\;\;\;\;\;\;\;\;\;\;\;\;\;\;\;\;\;v_{gluc}=uptake$$ (8)

This bi-level problem is reformulated as a single-stage optimization (see Step 1), yielding optimal objective coefficients ($c_{j}^{*}$) and an aligned flux distribution ($v_{j}^{*}$).


$$min{\left\|v_{j}-v_{j}^{exp}\right\|}_{\text{2}}^{\text{2}}\;$$

$${\text{s}.\text{t}.\;\;v}_{gluc}=uptake$$

$$\;\;\;\;\;\;\;\;\;\;\;\;\;\;\;\;\;\;\;\;\;\;\;\;\;\;\;\;\;\;\;\;\;\;\;\;\sum_{j}c_{j}^{*}v_{j}=v_{j=obj}=uptake\;.\;g$$

$$\;\;\;\;\;\;v_{j}\geq \text{0},\;\forall j\in M$$

$$\sum {\mathrm{\backslash nolimits}}_{j=1}^{M}S_{ij}v_{j}=0,\;\forall i\in N$$

$$\sum {\mathrm{\backslash nolimits}}_{i=1}^{N}u_{i}S_{ij}\geq 1,\;\;\forall j\in P$$

$$\sum {\mathrm{\backslash nolimits}}_{i=1}^{N}u_{i}S_{ij}\geq 0,\;\;\forall j\in M\cap \notin P\cap \notin gluc\;uptake$$

$$\;\;\sum {\mathrm{\backslash nolimits}}_{i=1}^{N}u_{i}S_{ij}+g\geq 0,\;\forall j\in gluc\;uptake$$ (9)

where $c_{j}^{*}$ is the objective coefficient vector with $c_{j}^{*}$ usually containing a 1 for the target reaction and 0s elsewhere, thus focusing the optimization on that specific reaction. Here, *u*_*i*_ represents the dual variable associated with the primal constraints, and *g* is the dual variable for the carbon source uptake constraint (e.g., glucose).

In the dual network representation, each reaction in the primal network becomes a node, and each metabolite becomes an edge, providing an alternative perspective on network dependencies. Mathematically, the calculation of minimum cut sets (MCs) in the dual network involves finding support-minimal vectors that satisfy the dual steady-state constraints. These support-minimal solutions are equivalent to elementary modes in the primal network [23] because they represent the smallest sets of reactions that allow a specific flux distribution without violating the steady-state or irreversibility constraints. Therefore, the optimized flux distribution ($v_{j}^{*}$) is then mapped using equations (2–4) to construct the Mass Flow Graph, which is based on the dual network [28] (see Step 2). Here, the dual problem validates the alignment of Coefficients of Importance with the network’s constraints [22,23,28]. By identifying MCs within the dual network, the framework determines essential pathways, quantifying each pathway’s contribution to the flux distribution and assessing the influence of external metabolites on network output. The normalized importance of each pathway with equations 5 and 6, represented by Coefficients of Importance as edge weights in the Mass Flow Graph, defines the updated objective reaction flux distribution.

## Supporting information

S1 Text Mass balance representation in metabolic network analysis. (PDF)

S2 Text Supplemental analyses and validation details. (PDF)

S3 Text Notation used in this study. (PDF)

S1 Data Experimental data utilized in this study. (XLSX)
