# Supplementary material for: Optimization-based framework with flux balance analysis (FBA) and metabolic pathway analysis (MPA) for identifying metabolic objective functions
Source: PLoS Comput Biol. 2025 Oct 27;21(10):e1013635. doi: 10.1371/journal.pcbi.1013635 (PMC12578352; doi:10.1371/journal.pcbi.1013635)
Supplement: S1 Text — (PDF) [file pcbi.1013635.s001.pdf]

S1 Text. Mass balance representation in metabolic network analysis.

The stoichiometric mass balance equation governing glucose metabolism in this study can be expressed as follows (Papoutsakis, 2000):

(2+a) = Glucose consumption

(2a−b−2k) = 0 = Pyruvate accumulation

(b−e−f−g−h−j) = 0 = Acetyl-CoA accumulation

(2a−1.75+d−2f−2g−i−2j) = 0 = $NADH_2$ formation

(b−c−d) = 0 = FdH2 accumulation

(e+g−f−h) = Acetate accumulation

(f−g) = Butyrate accumulation

c = H_2 accumulation

(b+h+2k) = CO_2 accumulation

g = Butanol accumulation

(h−i) = Acetone accumulation

i = Isopropanol accumulation

j = Ethanol accumulation

k = Acetoin accumulation

where a to k represent metabolic flux coefficients corresponding to different reaction pathways and metabolite production or consumption rates. These coefficients are derived from the stoichiometric matrix and reflect the distribution of metabolic fluxes in the network. The mass

balance accounts for key intermediate metabolites such as pyruvate, acetyl-CoA (AcCoA), $NADH_2$, and ATP, along with fermentation end products including acetate, butyrate, butanol, acetone, isopropanol, ethanol, and acetoin. The inclusion of FdH_2 and H_2 further captures redox balancing within the metabolic system.

Reference

Papoutsakis, E. T. (2000). Equations and calculations for fermentations of butyric acid bacteria. Biotechnology and Bioengineering, 67, 813-826.
