# Supplementary material for: Optimization-based framework with flux balance analysis (FBA) and metabolic pathway analysis (MPA) for identifying metabolic objective functions
Source: PLoS Comput Biol. 2025 Oct 27;21(10):e1013635. doi: 10.1371/journal.pcbi.1013635 (PMC12578352; doi:10.1371/journal.pcbi.1013635)
Supplement: S2 Text — (PDF) [file pcbi.1013635.s002.pdf]

S2 Text. Supplemental analyses and validation details.

This file contains additional results, figures, and model validation relevant to the main manuscript.

Section A. Flux Balance Analysis (FBA)

FBA is a mathematical method in computational biology used to model metabolic fluxes in biological systems. Linear equations are used to optimize reaction fluxes to achieve specific objectives. The basic FBA formula involves solving linear equations that represent the mass balance of each metabolite in the metabolic network. The equations are represented by a stoichiometric matrix (S) and a vector of fluxes (v):

$$Sv = 0 \qquad \textbf{Eq A1}$$

where S is an m x n matrix, where m is the number of metabolites in the network and n is the number of reactions, and v is an n-dimensional vector representing the fluxes of each reaction. The objective function, which represents the optimization goal, can be incorporated into the model by adding a constraint of the form:

$$c^T v = z \qquad \textbf{Eq A2}$$

where c is a vector representing the coefficients of the objective function, v is the flux vector, and z is the optimized value of the objective function. Once the set of linear equations has been constructed, it can be solved using linear programming techniques to obtain the optimal flux distribution that achieves the specified objective. The solution provides insights into the metabolic behavior of the system, such as the identification of key metabolic pathways and the prediction of optimal growth conditions. The convexity of FBA is contingent upon the particular problem formulation and the constraints incorporated into the model. In the context of our case study, both

the constraints and objective functions exhibit linearity, thereby fulfilling the requirements for convexity.

Section B. Mathematical Errors Involved Flux-dependent Weighted Graph

The flux-dependent weighted graph providing the overall scope of biosynthesis activities highly relies on the solutions obtained by FBA practice. Thus, the robustness and accuracy of FBA solutions play a crucial role in offering reliable explanations. The case study aims to define the issues of getting FBA solutions and understand the structure and properties of the system (Core *E. coli* Model as an example). The analysis should give us a sense of what is left to be improved and what kind of methodology can be applied in the case to deal with the impacts of the inherent stochastic and error that arise from mathematics during the FBA practice.

The goal is to predict the 95 reaction fluxes model in the system. To be more specific, there are 19 extracellular fluxes are generated: {'EX_ac(e)' 'EX_acald(e)' 'EX_akg(e)' 'EX_co2(e)' 'EX_etoh(e)' 'EX_for(e)' 'EX_fru(e)' 'EX_fum(e)' 'EX_glc(e)' 'EX_gln_L(e)' 'EX_glu_L(e)' 'EX_h(e)' 'EX_h2o(e)' 'EX_lac_D(e)' 'EX_mal_L(e)' 'EX_nh4(e)' 'EX_o2(e)' 'EX_pi(e)' 'EX_pyr(e)' 'EX_succ(e)'} , the expression is according to the open biological data source from KEGG, where “EX_(e)” is a common naming rule for representing cell excrete, and the name between two “_” is the abbreviation of one chemical. For example, etoh denotes ethanol. Among these products, some of the commonly seen chemicals can easily be measured by High-performance liquid chromatography (HPLC) in practice. Thus, there are seven products: {'EX_ac(e)' 'EX_etoh(e)' 'EX_fru(e)' 'EX_fum(e)' 'EX_glc(e)' 'EX_h2o(e)' 'EX_nh4(e)'} are measured (known) and serve as the lower bounds in the constraints (to relax the problem, since the measured data may not be true in the theoretical model). Based on the provided information,

we can formulate the question as the LP is shown as Eq A1**.** and Eq A2**.**, and the predicted fluxes are shown in **Figure B1 in S2 Text** by using Matlab 2022 linprog function with the biomass formation as the objective function.

To be noticed, there are two matrices for FBA, one is the original sparse stoichiometric matrix, and the other is reconstructed by singular value decomposition (SVD) to help us observe the difference of constructed flux-dependent graphs arising from the mathematical errors. SVD stands as a powerful mathematical technique with widespread applications across various fields. As a fundamental component of linear algebra, SVD decomposes a matrix into its constituent parts, yielding valuable insights into the underlying structure of the data. Notably, one of its key properties is the provision of the best low-rank approximation for a matrix, effectively reducing the reconstruction error when representing data in a lower-dimensional space.

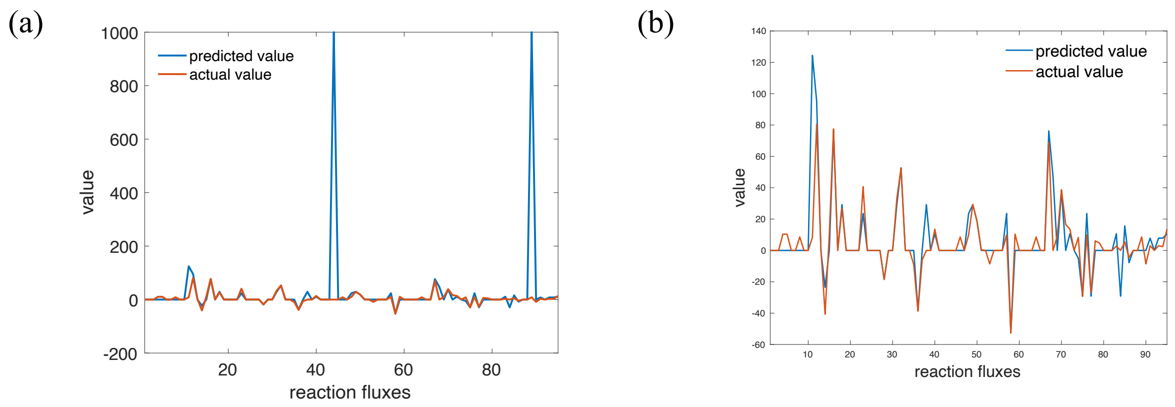

Figure B1 in S2 Text. The predicted fluxes (blue) and the actual fluxes value. (a) The 95 fluxes with the value range to 1000, obtained with FBA from the original stoichiometric matrix; (b) The actual fluxes and the predicted fluxes value with the reconstructed matrix obtained with SVD.

To quantify the performance of the prediction, the errors are calculated. The error is evaluated by the norm value of two vectors for the actual fluxes and the predicted fluxes, and it is returned as a scalar. The norm gives a measure of the magnitude of the elements:

$$\sum \sqrt{abs(v)^2} \qquad \textbf{Eq B1}$$

Applying **Eq B1.**, the total flux error for the FBA solution derived from the original stoichiometric matrix is approximately 1415.5. In contrast, the total flux error for the predicted flux values using the reconstructed matrix obtained with SVD is 143.8501, exhibiting a maximum absolute difference of 29.1550. Due to the discrepancies in the FBA solutions between the two scenarios, the flux-dependent weighted graph offers distinct interpretations. (**Figure B2 in S2 Text.**)

(a) (b)

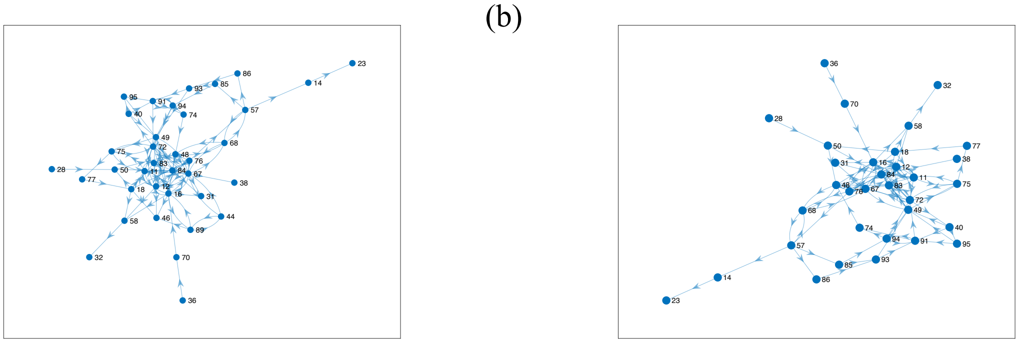

Figure B2 in S2 Text. The flux-dependent weighted graph, where the dot denotes the reactions in the stoichiometric matrix. (a) The flux-dependent weighted graph obtained with FBA solution; (b) flux-dependent weighted graph obtained with FBA solution of reconstructed SVD matrix.

The index of the nodes corresponding to Core E. coli model and fluxes table in S1 Data. For the graph derived from the original stoichiometric matrix (S), the characteristics are as follows:

Number of edges: 102; Number of nodes: 35. In comparison, the graph obtained from the reconstructed matrix displays the following features: Number of edges: 92; Number of nodes: 33. These differences in the number of edges and nodes between the two graphs indicate variations in the structure and connectivity of the flux-dependent weighted graphs when using the original S matrix and the reconstructed matrix.

The difference arising from mathematical approaches impacts the robustness of analysis and lack of consistency. To address this issue, we propose integrating the minimum-cut algorithm into metabolic network analysis for model simplification, thereby reducing the potential confusion arising from errors. By employing the minimum-cut algorithm, we can obtain similar minimal pathways of interest. For instance, when examining the pathways of glucose to carbon dioxide, the minimal pathways are illustrated in **Figure B3 in S2 Text.** and **Table B1 in S2 Text.**

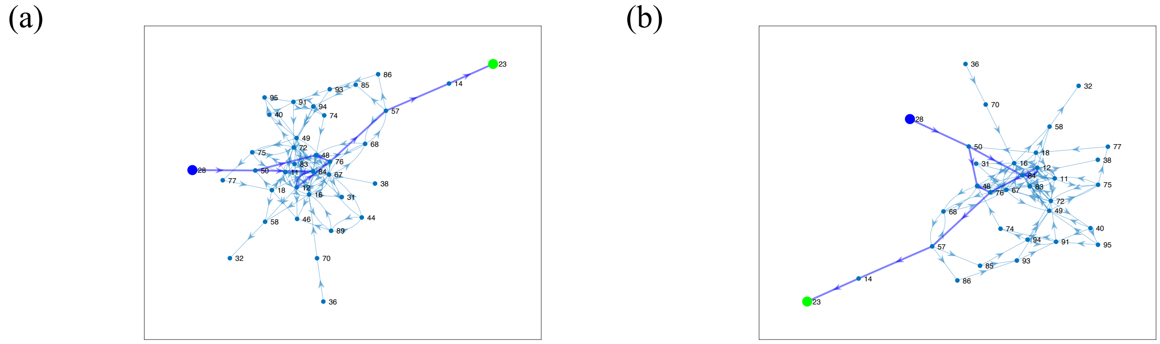

Figure B3 in S2 Text. The flux-dependent weighted graph, where the dot denotes the reactions in the stoichiometric matrix. (a) The flux-dependent weighted graph obtained with FBA solution; (b) flux-dependent weighted graph obtained with FBA solution of reconstructed SVD matrix.

Table B1 in S2 Text. Minimal Pathways of FBA solution obtained with a different matrix.

Minimal Pathway of FBA solution from the original matrix			Minimal Pathway of FBA solution from the reconstructed matrix		
**node s**	**node t**	**weight**	**node s**	**node t**	**weight**
'12'	'76'	3.55E-15	'12'	'76'	1.78E-14
'84'	'12'	3.55E-15	'84'	'12'	1.78E-14
'48'	'76'	18.5	'48'	'76'	18.5
'76'	'57'	18.5	'76'	'57'	18.5
'14'	'23'	18.5	'14'	'23'	18.5
'50'	'84'	3.55E-15	'50'	'84'	1.78E-14
'50'	'48'	18.5	'50'	'48'	18.5
'28'	'50'	18.5	'28'	'50'	18.5
'57'	'14'	18.5	'57'	'14'	18.5

From the results, it is evident that the same topology of the extracted minimal pathway is obtained from different flux-dependent graphs. Although there is a slight variation in the values of weights, it does not significantly impact the final interpretation.

By incorporating the minimum-cut algorithm into metabolic network analysis, as demonstrated in this case study, we can effectively identify crucial pathways and reaction modules with increased precision. This integration not only elevates the accuracy and efficiency of the research process but also plays a vital role in reinforcing the reliability and consistency of the performed analyses. As a result, it contributes significantly to advancing the understanding and exploration of complex metabolic networks across various scientific disciplines.

Section C. Variation of Coefficients of Importance (CoIs) in Response to Environmental Changes: Insights from E. coli Biomass Formation Preferences

The case examines an aerobic *Escherichia coli* system. In this case, CoIs are assigned to reaction fluxes associated with biomass production. It enables a comparison of metabolic network topologies. Additionally, biological insights in different growth conditions are highlighted using minimum cut sets (MCs) with minimal pathways that account for stoichiometry in metabolic networks.

Description of *E. coli* behavior in two different growth environments: The case explored the metabolic adjustments of *E. coli* in two different growth environments to compare their growth preferences [1]. The first environment involved aerobic growth in rich media with glucose (Gluc), while the second environment was aerobic growth in glucose with limited phosphate and ammonium (Gluc-lim). The *E. coli* model used in the study was a specific subset of the genome-scale metabolic reconstruction iAF1260 [2]. This model includes 72 metabolites and 95 reactions, with reversibility indicated as a Boolean value (1 denotes reversibility). The stoichiometric matrix, S, represents the relationships between metabolites and reactions and is a sparse matrix of size 72×95 . The reactions in the model are organized into 11 biochemical pathways responsible for carbon source breakdown, synthesis, and transformation for energy production and cellular maintenance (**Figure C1 A in S2 Text**) [3]. Under Gluc conditions, active glycolysis, pentose phosphate pathways, and ATP-driven processes contribute to biomass formation [3]. On the other hand, Gluc-lim conditions demand metabolic adaptations, including glycolysis, nutrient assimilation, aerobic metabolism, and nitrogen assimilation pathways, to produce vital cellular building blocks with reduced energy production efficiency. The metabolic network is accompanied by two vectors with a length of 190, representing the unfolded fluxes of forward and reverse

reactions under the conditions of sufficient glucose supply and limited ammonium and phosphate. The FBA solutions for the Gluc and Gluc-lim scenarios are provided, thus, Step 1 in the proposed framework was skipped. The FBA solutions are presented **in Figures C1 B** and **C1 C in S2 Text**, respectively, as flow diagrams, illustrating the corresponding fluxes of reactions (The graph displays only the top 10 fluxes, while a comprehensive list of all fluxes is provided in the Supporting Information.). However, the FBA solutions do not depict the flow distributions from their source to various destinations, and the inter-reaction directionalities remain unclear.

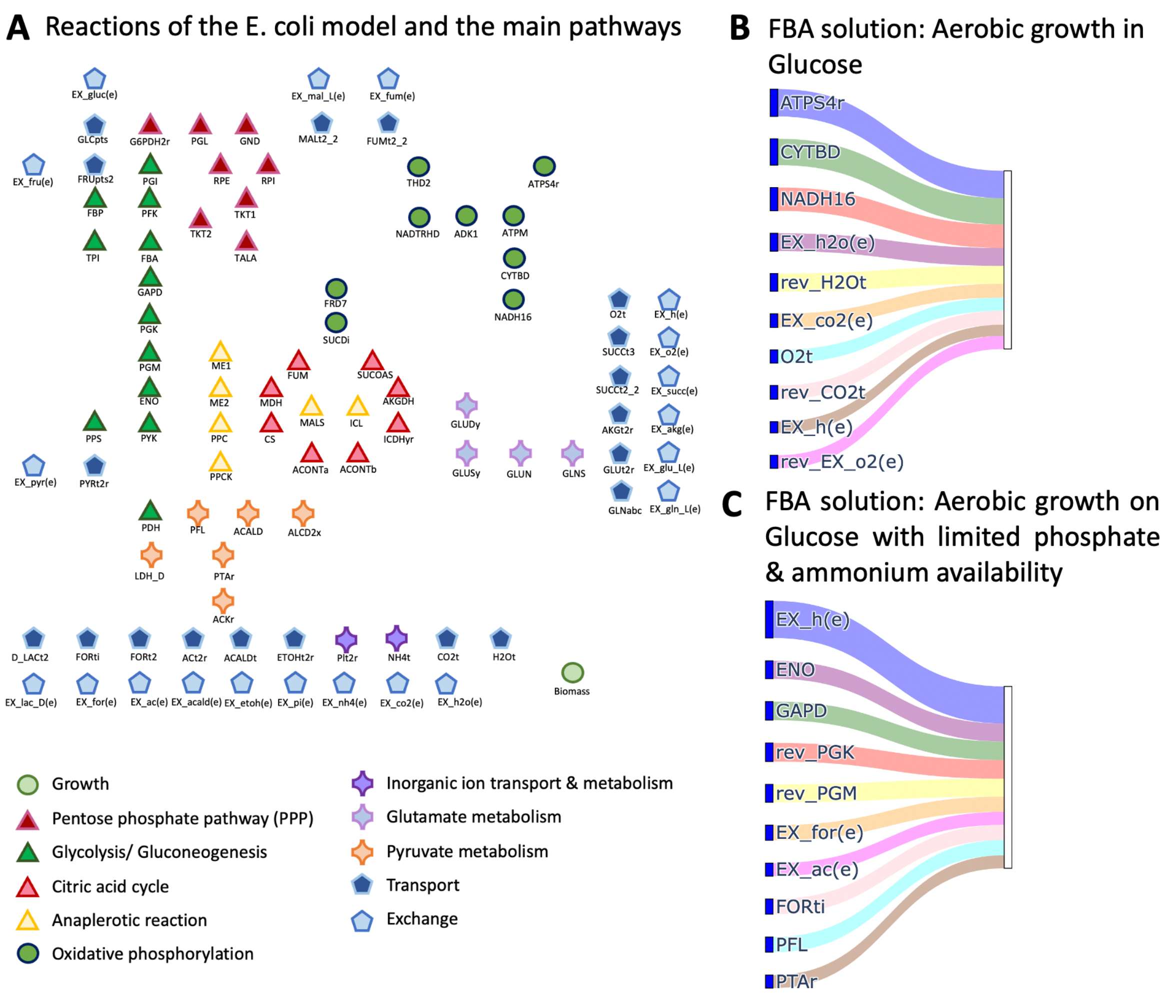

Figure C1 in S2 Text. A graphical representation of the *E. coli* model. (A) Categorization of *E. coli* metabolic model reactions into eleven fundamental biochemical pathways, including Growth, Pentose Phosphate Pathway (PPP), Glycolysis/Gluconeogenesis, Citric Acid Cycle, Anaplerotic Reactions, Oxidative Phosphorylation, Inorganic Ion Transport and Metabolism, Glutamate Metabolism, Pyruvate Metabolism, Transport, and Exchange processes. (B) the flow distribution of the FBA solutions for aerobic growth in a sufficient glucose condition. The graph includes only the top 10 fluxes. (C) the flow distribution of the FBA solutions for aerobic growth with glucose but limited the nutrients. The flow diagram's focus is on illustrating flow distribution rather than on connecting a single source to a target. The graph includes only the top 10 fluxes.

Flux Balance Analysis Results: When examining the FBA of *E. coli* under Gluc versus Gluc-lim, distinct metabolic shifts become evident (**Figure C1 B and C in S2 Text**). In glucose-rich conditions, the growth rate, represented by the flux Biomass_Ecoli_core_w_GAM, is nearly twice that observed under nutrient constraint. A vibrant TCA cycle, as indicated by the fluxes of AKGDH, SUCDi, and PDH, is present under glucose sufficiency but becomes less active under nutrient scarcity. This repression of the TCA cycle in limited conditions is counterbalanced by activation of overflow metabolism, evident from acetate secretion (EX_ac(e)) and the engagement of the pyruvate-formate lyase pathway (FORti and PFL). Furthermore, the significant reduction in the ATPS4r flux under nutrient limitation suggests a compromised energy generation. Oxygen consumption and the activity of the respiratory chain, represented by CYTBD and O2t fluxes, are also markedly reduced under these conditions. Moreover, the differential fluxes in rev_EX_co2(e) and rev_EX_nh4(e) offer insights into the uptake and

secretion changes of CO_2 and ammonia, respectively, under varying nutrient levels. In essence, *E. coli* exhibits metabolic adaptability, optimizing its growth and metabolic pathways in response to available resources. However, simply monitoring FBA fluxes does not fully capture the complex metabolic adaptations that *E. coli* exhibits under environment shifts (e.g., the importance of reactions within the network). The flux distributions and connectivity can offer a more insightful perspective. Therefore, a metabolic network topology analysis is performed next.

Mass Flow Graphs derived from Flux Balance Analysis solutions: The Mass Flow Graph (MFG) derived from FBA solutions are depicted in **Figure C2 A** and **Figure C3 in S2 Text.** In these graphs, the thickness of an edge line is directly proportional to the weight value of the connected edge (The interactive Sankey diagram, a flow diagram where arrow widths are proportional to the quantity they represent, displayed via the web interface, provides a visualization of the specific reaction flux weights within the Mass Flow Graph. The plot allows users to explore connections between reactions dynamically, highlighting flux distributions and key pathways.).

In the Gluc case, as shown in **Figure C1 A in S2 Text**, the significant flux from ATPS4r to NADH16 suggests efficient respiration and energy production. This flow illustrates the conversion of energy from ATP production to NADH utilization, a scenario typical of glucose-rich conditions. Here, cells can efficiently produce energy through oxidative phosphorylation, utilizing the flexibility metabolic in respiration. Conversely, the Gluc-lim case displays thinner connected edges in comparison to the Gluc case, signaling the constraints posed by nutrient limitation (**Figure C1 C in S2 Text**). The lack of nutrients necessitates metabolic adaptations and may invoke pathways like glycolysis, nutrient assimilation, aerobic metabolism, and nitrogen

assimilation. The aim is to synthesize essential cellular building blocks, even if it entails a compromise in energy production efficiency. The sequential fluxes spanning from GAPD to PGK and then to PGM underscore an active glycolysis. Under these nutrient constraints, cells exhibit overflow metabolism, which occurs when they take in more carbon than they can effectively process. Under such conditions, the TCA cycle and oxidative phosphorylation are less favorable pathways for cellular growth. Additionally, the flux transitions from GAPD to NADH16 and from NADH16 to EX_h(e) suggest that the cell maintains a certain level of respiratory activity, even if it's reduced compared to conditions where glucose is abundant. Mass Flow Graph enable us to determine the differences and identify key reactions between the two cases. However, these graphs, lacking specificity, often present a challenge in distinguishing between reaction shifts related to biomass formation and other reactions. For instance, in certain stress conditions, cells might ramp up their pentose phosphate pathway activity, leading to increased production of NADPH [4]. This shift could be a cellular response to counteract oxidative stress by producing more reducing power in the form of NADPH. While this is essential for the cell's survival and stress management, it might not have a direct influence on the primary biomass formation reactions [5]. Specifying the start and end reactions in topology analysis can help differentiate between these types of metabolic shifts.

Comparison of the Minimal Pathways: Figure C2 B and **Figure C B3 in S2 Text** depict the subnetworks, $P(s \rightarrow t)$, found by minimum-cut algorithm, where the graphs were specified with starting glucose flux (EX_glc(e)) to Biomass_Ecoli_core_w_GAM. In the glucose-rich scenario (**Figure C1 B in S2 Text.**), *E. coli* uptakes glucose (EX_glc(e) to GLCpts at the largest flow) and primarily channels it through the pentose phosphate pathway, as evidenced by the flux

GLCpts to G6PDH2r (the second large flow). G6PDH2r, involved in the pentose phosphate pathway, contributing to NADPH production [6]. Post the G6PDH2r reaction, fluxes diverge towards glycolysis (PYK) and aerobic respiration (CYTBD), both feeding biomass production. CYTBD functions in the electron transport chain, generating a proton gradient that drives ATP synthesis, providing energy for cellular processes, including cell growth [7]. Additionally, direct glucose flux contributes to biomass synthesis and the PDH reaction, bridging glycolysis and the TCA cycle. These pivotal metabolic reactions work together to optimize *E. coli*'s metabolism and support efficient biomass production under glucose-sufficient, aerobic conditions.

In contrary**, Figure C3 B in S2 Text** begins similarly with the initial steps from EX_glc(e) to GLCpts, but the subnetwork for Gluc-lim notably emphasizes the presence of pyruvate-formate lyase (PFL). This adaptation appears to be a strategy to cope with nutrient-limited conditions, facilitating survival and continued growth. The metabolic flux bifurcates towards the citric acid cycle, initiated by CS, and directs towards acetate production via PTAr. Within the citric acid cycle, intermediary reactions such as CS, ACONTa, ACONTb, and ICDHyr play roles in aiding in converting biomass for synthesis. Specifically, ACONTa and CS operate within the TCA cycle, delivering precursors essential for biosynthesis while also ensuring redox equilibrium [8]. Furthermore, reactions within the pentose phosphate pathway, including TALA and TKT2, highlight the importance of NADPH generation and ribose-5-phosphate synthesis, crucial for nucleotide production [6]. The presence of significant flux through the Acetate Kinase reaction (ACKr) suggests that, under these conditions, there might be an enhanced secretion of acetate, a characteristic of overflow metabolism to which the reaction plays a significant role in optimal energy production, maintain redox balance, or navigate metabolic constraints, ultimately supporting growth in nutrient-limited conditions [9].

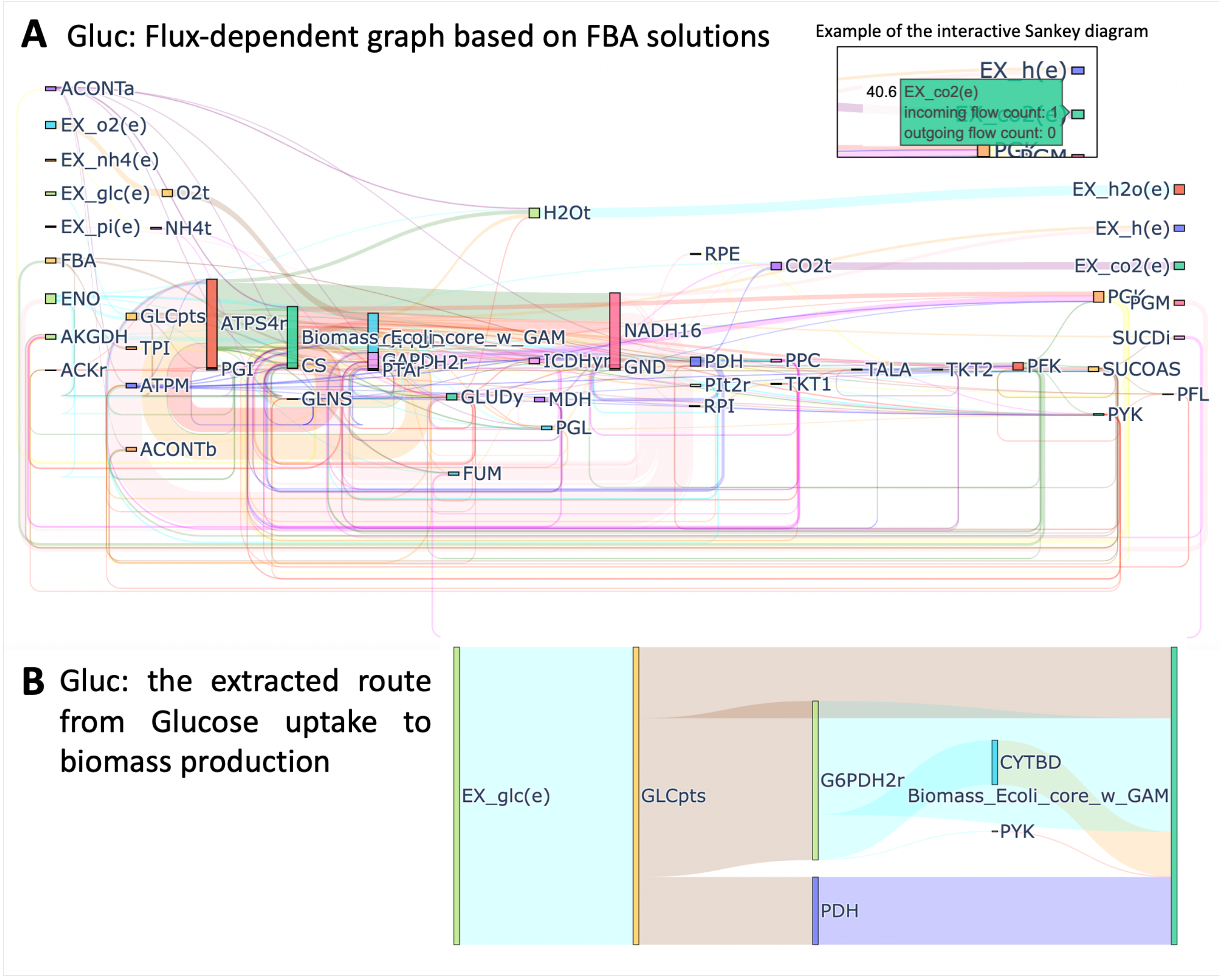

Figure C2 in S2 Text. The Mass Flow Graph. (A) the *E. coli* growth under aerobic growth in rich media in the glucose-rich case (Gluc). (B) The minimal pathway of the Mass Flow Graph from glucose uptake reaction to biomass formation for Gluc case. The minimal pathways do not contain loops and are in one direction from glucose uptake's reaction to biomass formation reaction. The graph details can be observed from the interactive Sankey diagram.

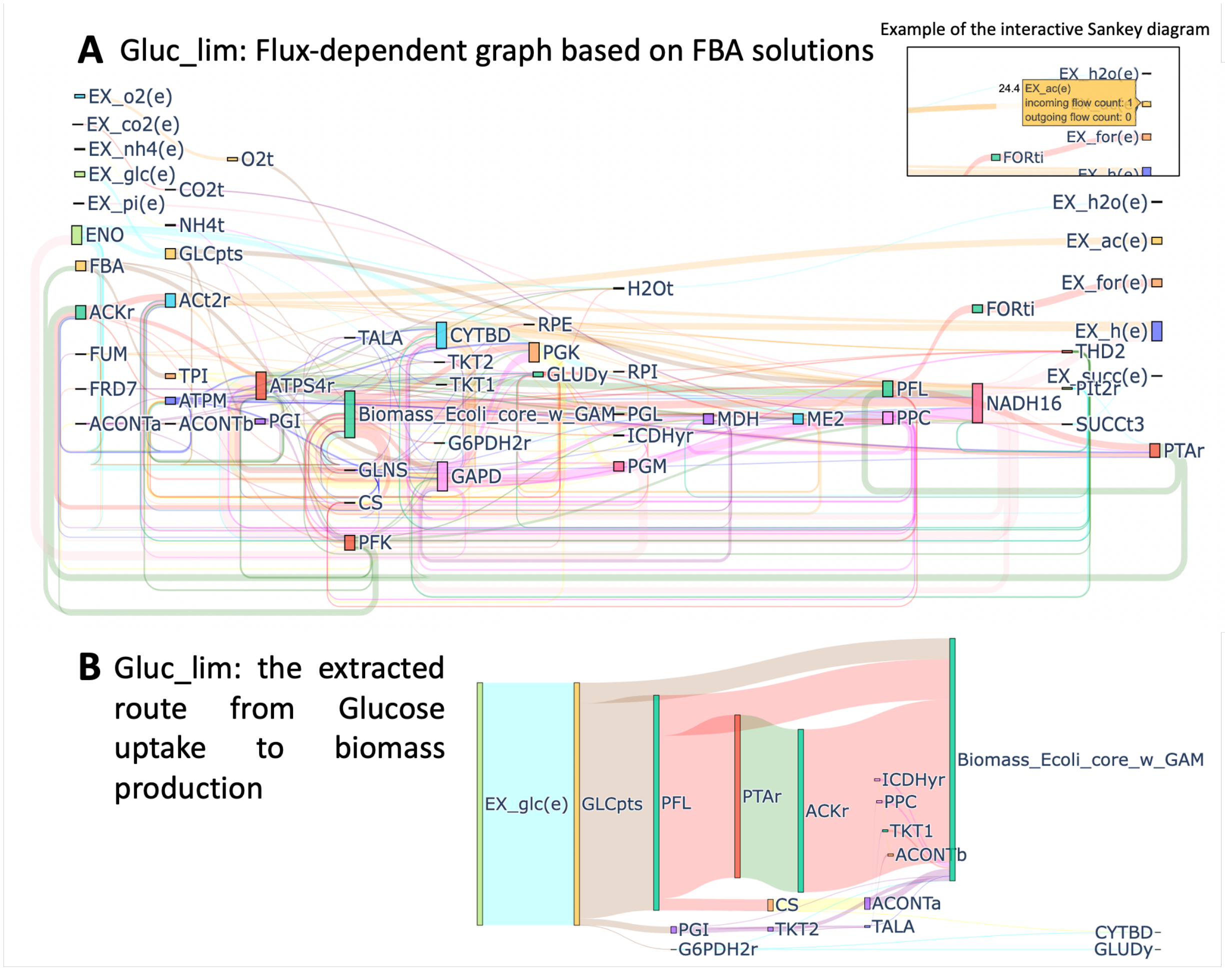

Figure C3 in S2 Text. The Mass Flow Graph. (A) the *E. coli* growth with glucose but limited the nutrients (Gluc-lim). (B) The minimal pathway of the Mass Flow Graph from glucose uptake reaction to biomass formation for Gluc-lim case. The line thickness corresponds to the value of the weights for graph. The graph details can be observed from the interactive Sankey diagram.

3.5 Comparison of the Coefficients of Minimal Pathways

When applying the minimum-cut algorithm to compare Mass Flow Graphs under different conditions, a higher cumulative weight for the inflow and outflow from the source to the sink indicates a more favorable environment for converting the starting metabolite into the desired

product. Conversely, a lower cumulative weight suggests reduced efficiency in this conversion. In scenarios with abundance source and sink metabolites and many low-weight intermediate reactions supporting the inflow and outflow, the resulting minimal pathways can contain a large number of edges. A quantitative comparison of pathway importance and relationships can be conducted using the criteria introduced in [10], refer as edge density. Edge density is computed as the sum of the weights of all edges within the minimal pathway divided by the total number of edges. The larger edge density represents a more favorable condition for biomass formation.

As shown in **Table C1 in S2 Text**, the higher total weight sum of the minimal pathway in the Gluc-lim case (Gluc-lim: 81.65 > Gluc: 53.90) reflects the complexity of the minimal pathway to sustain biomass formation. In contrast, the edge density (or average weights of $P(s \rightarrow t)$) for the minimal pathway under Gluc condition was higher than in the Gluc-lim case (Gluc:5.39 > Gluc-lim:2.81), indicating enhanced metabolic efficiency as larger weight per edge representing larger flow from glucose to the production of biomass. The results are further validated by examining the FBA solution (see DATA SET S1), which it was observed that the biomass flux in the Gluc case was 1.65, nearly twice as high as in the Gluc-lim case, where the biomass flux was 0.82.

Table C1 in S2 Text: The indicators correspond to the extracted minimal pathways of *E. coli* growth under two conditions: aerobic growth in rich media with glucose (Gluc case), and aerobic growth in glucose with limited phosphate and ammonium (Gluc-lim case).

	**Gluc**	**Gluc-lim**
Sum of weight	53.9025	81.6555
Average weight of minimal pathway	5.3902 (10 edges)	2.8157 (29 edges)

Previous studies using *Escherichia coli* flux data from isotopomer analysis revealed that biomass Coefficients of Importance remained similar under different conditions [11], despite significant differences in flux distributions. The **TIObjFind** framework, however, offers more insights of these variations. Under nutrient-limited conditions, *E. coli* reconfigures its metabolic priorities, with adjustments resulting in a smaller role for energy production and electron transport pathways. While the relative differences between FBA and TIObjFind CoIs for the two scenarios are generally similar, certain reactions: such as ATPS4r, CYTBD, NADH16, and GAPD show notable difference (see **Table C2 in S2 Text** and **Figure C4 in S2 Text**.). This discrepancy may arise because **TIObjFind** CoIs are calculated with respect to a defined source-to-target minimal pathway, where the flux of a reaction may be indirectly influenced by other metabolites or reactions. In contrast, FBA predicts flux distributions by globally optimizing an objective function, such as biomass production, under steady-state constraints across the entire network (e.g., biomass formation includes the combined contributions of precursor metabolites, energy production via ATP, and reducing power through NADH or NADPH formation). As a result, end reactions involving extracellular products, like CO_2, H_2O, and biomass, tend to align more closely with FBA predictions, as they directly reflect cumulative pathway outputs. The alignment of the relative difference of the end reactions (e.g., the synthesis of pathway end-products) in both approaches highlights the shared importance of terminal objectives. This alignment underscores **TIObjFind**'s ability to deconstruct pathway-level behavior, which is particularly useful in scenarios where localized pathway-specific insights are needed, rather than a system-wide optimization.

Table C2 in S2 Text. Comparison of relative differences in reaction fluxes under glucose-rich (Gluc) and glucose-limited (Gluc-lim) conditions using FBA and the relative differences in Coefficients of Importance from **TIObjFind**.

**Reaction**[a]	**FBA (Gluc)**	**FBA (Gluc-lim)**	**FBA relative difference**	**TIObjFind**[b] **relative difference**
ATPS4r	80.61	16.48	79.56%	37.35%
Biomass	1.65	0.83	50.00%	47.76%
CS	10.37	0.89	91.40%	87.89%
CYTBD	77.48	23.55	69.61%	32.87%
EX_ac(e)	0.00	24.43	Na	Na
EX_co2(e)	40.65	0.00	100.00%	100.00%
EX_h2o(e)	52.69	4.54	91.38%	80.79%
NADH16	68.90	23.56	65.81%	35.33%
GAPD	29.31	34.80	18.73%	42.34%
ACONTa	10.37	0.89	91.40%	91.40%
ACONTb	10.37	0.89	91.40%	93.50%

[a] abbreviation of reactions.

[b] The relative difference in the average weight of the minimal pathway between the Gluc and Gluc-lim scenarios.

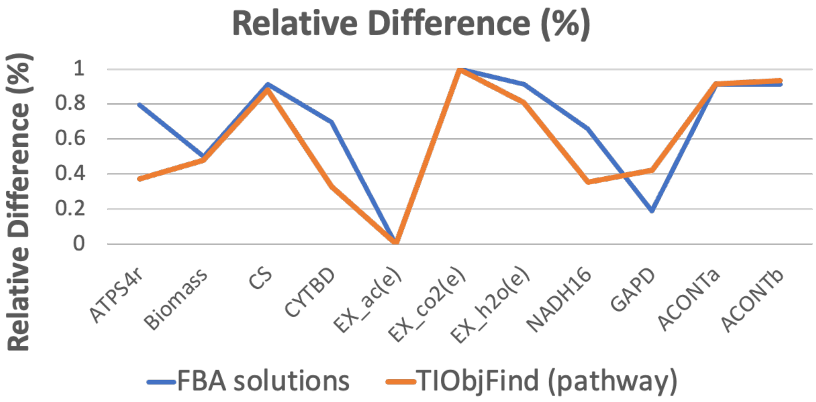

Figure C4 in S2 Text. Comparison of relative differences (%) in reaction fluxes between glucose-rich (Gluc) and glucose-limited (Gluc-lim) conditions as predicted by Flux Balance Analysis (FBA) solutions and the **TIObjFind** framework. The reactions shown include ATPS4r, Biomass, CS, CYTBD, EX_ac(e), EX_co2(e), NADH16, GAPD, and ACONT (a and b variants). The FBA solutions and **TIObjFind** pathway weights reveal distinct patterns in how these reactions adapt under different nutrient conditions.

Section D. Sensitivity and Validation Analyses of TIObjFind

Sensitivity Analysis of Cofactor/Intermediate Scaling Constants: In the baseline weighting scheme (Section B), scaling constants of 0.1 for key intermediates (pyruvate, acetyl-CoA) and 0.01 for redox cofactors ($NADH_2$, FdH_2) were adopted from prior studies, reflecting their relative importance in maintaining the pseudo-steady-state balances in the stoichiometric model ($S \cdot v = 0$). To examine the robustness of this assumption, we increased these constants by 50% and 100% and re-calculated flux predictions using both (ii) normalized experimental values and (iii) TIObjFind-derived weights. Results for EXP1 and EXP2 are summarized in **Tables D1 and D2 in S2 Text** and **Figures D1 and D2 in S2 Text.**

For EXP1, the modified scaling factors slightly tightened the residual minimization and led to marginally better agreement between predicted and experimental fluxes, reducing overall prediction error. This suggests that increasing the numerical weights reduced the relaxation of pseudo-steady-state constraints, thereby improving the fit. For EXP2, the prediction error increased modestly, with deviations of up to ~8% for certain metabolites. Nonetheless, the overall predictions remained within an acceptable error range, and the main product trends were preserved. Importantly, in both experiments, the CoI profiles and pathway prioritization remained stable, indicating that the method is not qualitatively sensitive to moderate changes in redox/intermediate scaling constants.

Table D1 in S2 Text. Predicted metabolite yields for EXP1 under baseline, +50%, and +100% scaling of pyruvate, acetyl-CoA, $NADH_2$, and FdH_2 weights. Two weighting strategies are compared: (ii) normalized experimental values and (iii) TIObjFind-derived weights. Experimental yields are reported in mol per 100 mol glucose.

	EXP1	Calculated					
		baseline		increase 50%		increase 100%	
		using exp as weight (ii)	TIObjFind weight (iii)	using exp as weight (ii)	TIObjFind weight (iii)	using exp as weight (ii)	TIObjFind weight (iii)
Butanol	29.30	30.23	29.26	30.07	29.22	29.91	29.30
acetone	9.95	9.80	9.96	9.83	9.96	9.85	9.95
ethanol	14.65	14.68	14.65	14.67	14.64	14.66	14.65
acetate	24.01	23.19	24.02	23.22	24.03	23.30	24.01
butyrate	36.50	35.69	36.49	35.58	36.49	35.58	36.50
H2	174.65	171.68	174.14	171.49	173.57	171.24	174.65
CO2	212.11	211.17	211.46	210.13	210.73	209.24	212.11
isopropanol	0.00	0.00	0.00	0.00	0.00	0.00	0.00

acetoin	5.40	5.40	5.40	5.40	5.40	5.40	5.40

Figure D1 in S2 Text. Relative prediction error (%) for EXP1 when redox/intermediate weights (pyruvate, acetyl-CoA, $NADH_2$, FdH_2) are increased by 50% and 100%, compared to baseline.

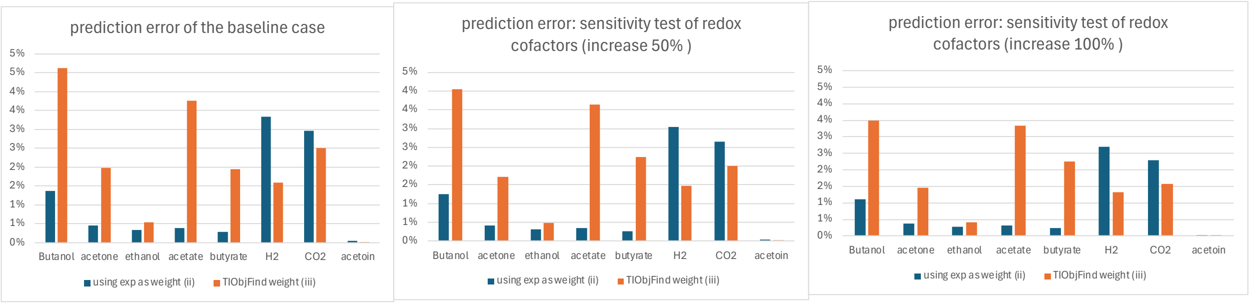

Table D2 in S2 Text. Predicted metabolite yields for EXP2 under baseline, +50%, and +100% scaling of pyruvate, acetyl-CoA, $NADH_2$, and FdH_2 weights. Two weighting strategies are compared: (ii) normalized experimental values and (iii) TIObjFind-derived weights.

	EXP2	Calculated					
		baseline		increase 50%		increase 100%	
		using exp as weight (ii)	TIObjFind weight (iii)	using exp as weight (ii)	TIObjFind weight (iii)	using exp as weight (ii)	TIObjFind weight (iii)
Butanol	10.42	10.47	10.69	11.35	10.67	11.33	10.42
acetone	-3.13	-3.03	-0.01	-0.01	-0.01	-0.01	-3.13
ethanol	13.61	13.62	13.64	13.66	13.63	13.66	13.61
acetate	30.70	30.58	30.67	30.45	30.67	30.48	30.70
butyrate	54.21	54.74	54.16	54.46	54.14	54.50	54.21
H2	187.38	186.74	189.29	187.21	189.09	187.15	187.38
CO2	194.17	193.84	193.70	193.61	193.58	193.67	194.17
isopropanol	6.61	6.05	3.33	2.68	3.41	2.74	6.61
acetoin	5.10	5.10	5.10	5.10	5.10	5.10	5.10

Figure D2 in S2 Text. Relative prediction error (%) for EXP2 when redox/intermediate weights are increased by 50% and 100%, compared to baseline.

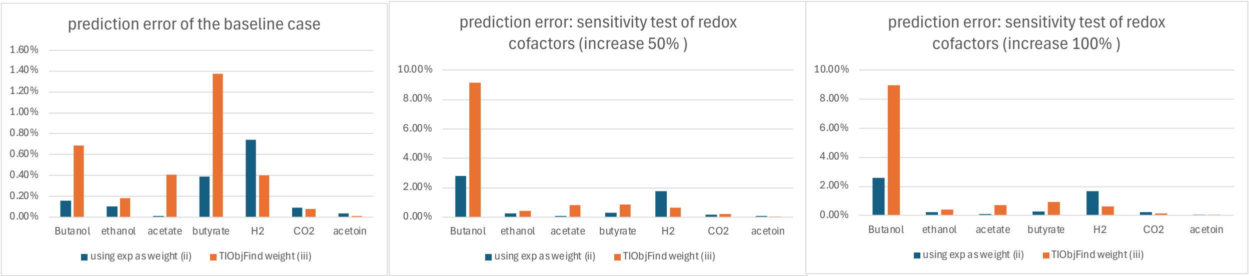

Validation of TIObjFind with Alternative Metabolite Sets: To further evaluate the robustness of TIObjFind and to rule out potential inflation of prediction accuracy, an additional validation was performed using alternative metabolite sets. In this analysis, selected extracellular metabolites (butanol, acetone, ethanol, isopropanol, and acetoin) were included in the fitting set, whereas intermediates such as acetate, butyrate, H_2, and CO_2 were excluded from the fit and instead reserved for validation.

Tables D3 and D4 in S2 Text present the results for EXP1 and EXP2, respectively. In both cases, TIObjFind maintained strong predictive accuracy for the excluded validation set, with errors generally below 5% and within ~8% for acetate and butyrate. Importantly, TIObjFind provided more reliable predictions for redox-related byproducts (H_2, CO_2) compared with unweighted or normalized weighting schemes, indicating that the estimated Coefficients of Importance capture meaningful pathway-level tradeoffs even when metabolites are withheld from the fitting stage. This analysis demonstrates that TIObjFind's ability to flexibly incorporate different metabolite sets depending on data availability further illustrates the practical applicability of the framework.

Table D3 in S2 Text. EXP1 validation of TIObjFind using different metabolite sets. Metabolites marked with "a" were included in the fitting set; all others were reserved for validation.

	experiment	Calculated					
	EXP1	no weight (i)	using exp as weight (ii)	TIObjFind weight (iii)	no weight (i)	using exp as weight (ii)	TIObjFind weight (iii)
Butanol	28.90	28.90 [a]	28.90 [a]	28.90 [a]	28.90 [a]	28.90 [a]	28.90 [a]
acetone	10.00	10.00[a]	10.00 [a]	10.00 [a]	10.00 [a]	10.00 [a]	10.00 [a]
ethanol	14.60	14.60 [a]	14.60 [a]	14.60 [a]	14.60 [a]	14.60 [a]	14.60 [a]
acetate	24.10	25.05	25.05	24.95	24.10 [a]	24.10 [a]	24.10 [a]
butyrate	36.40	36.40 [a]	36.40 [a]	36.40 [a]	36.88	36.91	36.87
H2	169.00	177.20	177.19	177.35	176.25	176.45	176.26
CO2	206.00	212.80	212.80	213.05	212.80	213.00	212.81
isopropanol	0.00	0.00 [a]	0.00 [a]	0.12 [a]	0.00 [a]	0.04 [a]	0.00 [a]
acetoin	5.40	5.40 [a]	5.40 [a]	5.40 [a]	5.40 [a]	5.40 [a]	5.40 [a]

[a] data used in model calculation

Table D4 in S2 Text. EXP2 validation of TIObjFind using different metabolite sets. Metabolites marked with "a" were included in the fitting set; all others were reserved for validation.

		Calculated					
	EXP2	no weight (i)	using exp as weight (ii)	TIObjFind weight (iii)	no weight (i)	using exp as weight (ii)	TIObjFind weight (iii)
Butanol	10.40	10.40 [a]	10.40 [a]	10.40 [a]	10.40 [a]	10.40 [a]	10.40 [a]
acetone	0.00	0.00 [a]	0.00 [a]	0.00 [a]	0.00 [a]	-0.01 [a]	-0.01 [a]
ethanol	13.60	13.60 [a]	13.60 [a]	13.60 [a]	13.60 [a]	13.60 [a]	13.60 [a]
acetate	30.70	38.20	38.16	38.16	30.70 [a]	30.70 [a]	30.70 [a]
butyrate	54.00	54.00 [a]	54.00 [a]	54.00 [a]	57.75	57.76	57.72
H2	186.00	194.60	194.57	194.58	187.10	187.08	187.36
CO2	194.00	190.80	190.82	190.82	190.80	190.79	191.07
isopropanol	0.00	0.00 [a]	0.02 [a]	0.02 [a]	0.00 [a]	0.00 [a]	0.12 [a]
acetoin	5.10	5.10 [a]	5.10 [a]	5.10 [a]	5.10 [a]	5.10 [a]	5.10 [a]

[a] data used in model calculation

4.3 Case Study 1: Intracellular Flux Estimates from Different Weighting Strategies

For both experiments, all methods reproduce near-zero fluxes for redox cofactors, consistent with the lumped structure of the model. Pyruvate and AcCoA predictions vary more noticeably, with TIObjFind providing intermediate estimates that avoid the over- or under-estimation observed under no-weight and direct-weighting schemes. This highlights that, while Case Study 1 is not strongly informative for intracellular flux differences, TIObjFind offers more stable and interpretable CoI assignments, which becomes important in larger, non-lumped networks.

Table D5 in S2 Text. Predicted intracellular fluxes (mol/100 mol glucose) in Case Study 1 under different weighting strategies.

Metabolite	Exp1 (b)	No weight	Using data	TIObjFind	Exp2 (b)	No weight	Using data	TIObjFind
Glucose	99.4	99.4	99.4	99.4	93.4	93.4	93.4	93.4
Pyruvate	0	2.34	0.64	1.43	0	-0.59	0.10	-0.02
AcCoA	0	1.70	1.19	1.24	0	-0.04	-0.01	-0.05
$NADH_2$	0	0.22	0.007	0.004	0	0.29	0.001	0.001
FdH_2	0	0.22	0.007	0.004	0	0.29	0.001	0.001

Section E. Validation of TIOBJFIND-Derived Objectives Against 13C-metabolic flux analysis (MFA) Fluxes

To assess the consistency of our TIObjFind framework with experimental metabolic flux data, we constructed a mass-flow graph (MFG) representation of *Clostridium acetobutylicum* central metabolism using the published ^{13}C-MFA best-fit flux distribution from the minimal network model published by Au et al. (2014). [12] These best-fit values are obtained from ^{13}C metabolic flux analysis (MFA) and serve as reference flux distributions.

The stoichiometric model (92 metabolites × 81 reactions) was aligned with the 81 reported best-fit fluxes, allowing direct use of the ^{13}C-MFA values to initialize edge weights for the MFG. Application of the minimum-cut algorithm identified pathway flows from the source (glucose uptake via hexokinase, ~100) to multiple sinks (butanol, butyrate, acetone, ethanol, and acetate). The resulting max-flow values aligned exactly with the reported MFA best-fit secretion fluxes (Table E1 in S2 Text).

To further validate predictive capacity, we derived relative objective weights from the TIOBJFIND procedure and performed a weighted FBA optimization under tight ^{13}C constraints (±5% around best-fit values; glucose phosphorylation fixed at 99.9–100.1). The optimized FBA solution reproduced product secretion fluxes within 5% relative error of the MFA targets (**Table E2 in S2 Text**). These results demonstrate that the TIOBJFIND-derived objective formulation, when constrained by ^{13}C-MFA measurements, is able to recover experimentally validated pathway flows.

Figure E1 in S2 Text. Mass-flow graph representation of *C. acetobutylicum* flux distribution. Glucose uptake (source) is distributed toward butanol, butyrate, acetone, ethanol, and acetate (sinks). Edge thickness indicates flux magnitude; highlighted paths correspond to min-cut partitions.

from	to	weight
"Gluc.Ext + ATP -> G6P + ADP"	"G6P <=> F6P (net)"	14.953
"Gluc.Ext + ATP -> G6P + ADP"	"GAP + NAD + ADP <=> 3PG + ATP + NADH (net)"	34.422
"Gluc.Ext + ATP -> G6P + ADP"	"BtCoA + ADP -> Bt + ATP"	15.226
"G6P <=> F6P (net)"	"F6P + ATP -> FBP + ADP"	14.953
"F6P + ATP -> FBP + ADP"	"BtCoA + ADP -> Bt + ATP"	14.953
"GAP + NAD + ADP <=> 3PG + ATP + NADH (net)"	"SucCoA + ADP <=> Suc + ATP (net)"	0.17043
"GAP + NAD + ADP <=> 3PG + ATP + NADH (net)"	"Pyr + CO2 + ATP -> OAC + ADP"	0.83648
"GAP + NAD + ADP <=> 3PG + ATP + NADH (net)"	"AcAcCoA + NADPH + NADH -> BtCoA + NADP + NAD"	33.415
"SucCoA + ADP <=> Suc + ATP (net)"	"BtCoA + ADP -> Bt + ATP"	0.17043
"Pyr + CO2 + ATP -> OAC + ADP"	"BtCoA + ADP -> Bt + ATP"	0.83648
"AcAcCoA + NADPH + NADH -> BtCoA + NADP + NAD"	"BtCoA + ADP -> Bt + ATP"	33.415
"BtCoA + ADP -> Bt + ATP"	"Bt -> Bt.Ext"	64.601

Table E1 in S2 Text: Comparison of MFG-derived max-flow values and ^{13}C-MFA best-fit secretion fluxes. Perfect alignment is observed for all major products.

**Sink**	**MFG max-flow**	**^{13}C MFA best-fit**
Butanol	5.0718	5.0718
Butyrate	64.601	64.601
Acetone	2.2874	2.2874
Ethanol	1.829	1.829
Acetate	30.575	30.575

Table E2 in S2 Text. Weighted FBA prediction versus ^{13}C-MFA best-fit fluxes (tight ±5% bounds). Prediction error across all sinks is within 5%.

**sink**	**v_FBA**	**^{13}C best-fit**
**BtOH**	5.3254	5.0718
**Butyrate**	67.8311	64.6010
**Acetone**	2.4018	2.2874
**Ethanol**	1.9204	1.8290
**Acetate**	32.1037	30.5750

Section F. Sink Metabolite Selection Robustness Test

To evaluate the impact of including additional fermentation products as sinks, we recomputed CoI distributions with butyrate and acetone added alongside the four primary products. The results (**Table F1 in S2 Text**) show modest reweighting but no change in the relative dominance of butanol, isopropanol, acetate, and ethanol across fermentation stages. Acetoin and 2,3-butanediol were excluded due to near-zero or consistently low fluxes (< 2 mM). These findings support the robustness of TIObjFind to sink selection, while also underscoring the limited interpretability of low-flux or volatile products such as acetone.

Table F1 in S2 Text. CoI distributions with extended sink set (values normalized).

	t=2.0 hr	t=11.9 hr	t= 19.3 hr	t=30.3 hr
BuOH	0.09	0.14	0.16	0.24
IPA	0.13	0.18	0.20	0.56
acetate	0.35	0.14	0.16	0.00
ethanol	0.16	0.19	0.21	0.08
but	0.19	0.19	0.11	0.05
acetone	0.09	0.17	0.17	0.07

Table E2 in S2 Text. Fermentation product titers over time: experimental data and prediction data.

Acetoin and 2,3-butanediol remained below 2 mM throughout.

TIObjFind-obj FBA					
	t=0	t=11.3	t=30.3	t=40.3	t=49.8
isopropanol	0	1.2845	10.6478	12.0404	12.692
acetate	0.491	2.2832	3.804	3.5765	3.3183
butanol	0	0.3109	7.8166	7.8952	8.5651
ethanol	0.9269	0.9455	1.9731	2.1076	2.1951
acetone	1.03E-07	0.1678	1.4859	0.7269	0.4898
butyrate	0.0564	1.4234	1.6076	1.6208	1.7052
sample point					
	t=0	t=11.3	t=30.3	t=40.3	t=49.8
isopropanol	0	1.2759	10.6383	12.0458	12.6967
acetate	0.472	2.2793	3.7947	3.5659	3.3071
butanol	0	0.3083	7.8161	7.8969	8.5706
ethanol	0.9269	0.9454	1.9723	2.1072	2.1957
acetone	0	0.169	1.4863	0.7248	0.4896
butyrate	0.0564	1.9049	2.0882	2.1014	2.186

Abbreviations

Gluc: *E. coli* aerobic growth in rich media with glucose

Gluc-lim: *E. coli* aerobic growth in glucose with limited phosphate and ammonium

TCA: tricarboxylic acid

AKGDH: 2-Oxoglutarate dehydrogenase

SUCDi: succinate dehydrogenase

ATPS4r: ATP synthase (four protons for one ATP)

ACKr: acetate kinase CS: citrate synthase

ACONTa: aconitase (half-reaction A, Citrate hydro-lyase)

ACONTb: aconitase (half-reaction B, Isocitrate hydro-lyase)

PFL: pyruvate-formate lyase

CYTBD: cyl chrome oxidase bd (ubiquinol-8: 2 protons)

Biomass_Ecoli_core_W_GAM: Biomass Objective Function with GAM

EX_xxx(e): secrete/ or uptake metabolites to/ or from the extracellular space.

Reference

(1) Orth, J. D.; Fleming, R. M. T.; Palsson, B. Ø. Reconstruction and Use of Microbial Metabolic Networks: the Core *Escherichia coli* Metabolic Model as an Educational Guide. *EcoSal Plus* **2010**, *4* (1), ecosalplus.10.12.11. DOI: 10.1128/ecosalplus.10.2.1 (acccessed 2023-04-03 16:47:55).DOI.org (Crossref).
(2) Feist, A. M.; Zielinski, D. C.; Orth, J. D.; Schellenberger, J.; Herrgard, M. J.; Palsson, B. Ø. Model-driven evaluation of the production potential for growth-coupled products of Escherichia coli. *Metabolic Engineering* **2010**, *12* (3), 173-186. DOI: 10.1016/j.ymben.2009.10.003 (acccessed 2023-04-03 17:14:15).DOI.org (Crossref). Orth, J. D.; Conrad, T. M.; Na, J.; Lerman, J. A.; Nam, H.; Feist, A. M.; Palsson, B. Ø. A comprehensive genome-scale reconstruction of *Escherichia coli* metabolism—2011. *Molecular Systems Biology* **2011**, *7* (1), 535. DOI: 10.1038/msb.2011.65 (acccessed 2023-04-03 17:13:51).DOI.org (Crossref).
(3) Beguerisse-Díaz, M.; Bosque, G.; Oyarzún, D.; Picó, J.; Barahona, M. Flux-dependent graphs for metabolic networks. *npj Systems Biology and Applications* **2018**, *4* (1), 32. DOI: 10.1038/s41540-018-0067-y (acccessed 2023-04-03 15:40:15).DOI.org (Crossref).
(4) Christodoulou, D.; Link, H., Fuhrer, T. ; Kochanowski, K.; Gerosa, L.; Sauer, U. Reserve flux capacity in the pentose phosphate pathway enables Escherichia coli's rapid response to oxidative stress. *Cell systems* **2018**, *6* (5), 569-578.
(5) Stincone, A.; Prigione, A.; Cramer, T.; Wamelink, M. M.; Campbell, K.; Cheung, E.; Ralser, M. The return of metabolism: biochemistry and physiology of the pentose phosphate pathway. *Biological Reviews* **2015**, *90* (3), 927-963. DOI: https://doi.org/10.1111/brv.12140.
(6) Hinton, H. S. E.coli Core Model for Beginners (PART 3). http://gibbs.unal.edu.co/cobradoc/cobratoolbox/tutorials/reconstruction/ecoliCoreModel/part3/iframe_tutorial_ecoliCoreModel_part3.html (accessed 2023-02-20).
(7) Xia, X.; Wu, S.; Li, L.; Xu, B.; Wang, G. The Cytochrome bd Complex Is Essential for Chromate and Sulfide Resistance and Is Regulated by a GbsR-Type Regulator, CydE, in

Alishewanella Sp. WH16-1. *Frontiers in Microbiology* **2018**, *9*, 1849. DOI: 10.3389/fmicb.2018.01849 (acccessed 2023-04-03 17:40:33).DOI.org (Crossref).
(8) Kang, W.; Suzuki, M.; Saito, T.; Miyado, K. Emerging Role of TCA Cycle-Related Enzymes in Human Diseases. *International Journal of Molecular Sciences* **2021**, *22* (23), 13057. DOI: 10.3390/ijms222313057 (acccessed 2023-04-14 17:02:31).DOI.org (Crossref). Consortium, U. UniProt: the Universal Protein Knowledgebase in 2023. https://www.uniprot.org/uniprotkb/P25516/entry (accessed 2023-04-01).
(9) Vemuri, G. N.; Altman, E.; Sangurdekar, D. P.; Khodursky, A. B.; Eiteman, M. Overflow metabolism in Escherichia coli during steady-state growth: transcriptional regulation and effect of the redox ratio. *Applied and environmental microbiology* **2006**, *72* (5), 3653-3661. DOI: https://doi.org/10.1128/AEM.72.5.3653-3661.2006.
(10) Wen, C.-M.; Yan, Z.; Liang, Y.-C.; Wu, H.; Zhou, L.; Yao, Y. A control chart-based symbolic conditional transfer entropy method for root cause analysis of process disturbances. *Computers & Chemical Engineering* **2022**, *164*, 107902. DOI: 10.1016/j.compchemeng.2022.107902 (acccessed 2023-04-03 18:30:53).DOI.org (Crossref).
(11) Burgard, A. P.; Maranas, C. D. Optimization-based framework for inferring and testing hypothesized metabolic objective functions. *Biotechnology and bioengineering* **2003**, *82* (6), 670-677.
(12) Au, J.; Choi, J.; Jones, S. W.; Venkataramanan, K. P.; Antoniewicz, M. R. Parallel labeling experiments validate Clostridium acetobutylicum metabolic network model for 13C metabolic flux analysis. *Metabolic engineering* **2014**, *26*, 23-33.
