# Supplementary material for: Optimization-based framework with flux balance analysis (FBA) and metabolic pathway analysis (MPA) for identifying metabolic objective functions
Source: PLoS Comput Biol. 2025 Oct 27;21(10):e1013635. doi: 10.1371/journal.pcbi.1013635 (PMC12578352; doi:10.1371/journal.pcbi.1013635)
Supplement: S3 Text — (PDF) [file pcbi.1013635.s003.pdf]

S3 Text. Notation used in this study.

Notation

S: stoichiometric matrix

S_{2m}: The unfolded version of the stoichiometric matrix

i: metabolite, i = 1, …,n

j: reaction j = 1, …,m

c^{obj}: the vector of Coefficients of Importance, the elements ${c_j}^{obj}$ sum up to 1

v: FBA solution vector

Z: Objective function, where $Z = c^{obj} \cdot v$

$\mathcal{G}(V, E)$: with a source node s, a sink node t, and a set of edge capacities

$\mathcal{C}(j, j')$: the edge capacities

E: the edge sets, where $(j, j') \in E$

V: the vertex sets, $j \in V$

$w_{j,j'}$ is the weight of edge (j, j')

$MCs^{s \to t}$: the minimum cut sets that disjoint sets $\mathcal{S}$ and T, where $s \in \mathcal{S}$ and $t \in T$

$P(s \to t)$: minimal pathway from node s to t
